# A yellow fever virus NS4B inhibitor not only suppresses viral replication, but also enhances the virus activation of RIG-I-like receptor-mediated innate immune response

**DOI:** 10.1371/journal.ppat.1010271

**Published:** 2022-01-21

**Authors:** Zhao Gao, Xuexiang Zhang, Lin Zhang, Shuo Wu, Julia Ma, Fuxuan Wang, Yan Zhou, Xinghong Dai, Esther Bullitt, Yanming Du, Ju-Tao Guo, Jinhong Chang

**Affiliations:** 1 Baruch S. Blumberg Institute, Doylestown, Pennsylvania, United States of America; 2 Bioinformatics and Biostatistics Facility, Fox Chase Cancer Center, Philadelphia, Pennsylvania, United States of America; 3 Department of Physiology and Biophysics, Case Western Reserve University, School of Medicine, Cleveland, Ohio, United States of America; 4 Department of Physiology & Biophysics, Boston University School of Medicine, Boston, Massachusetts, United States of America; Thomas Jefferson University - Center City Campus: Thomas Jefferson University, UNITED STATES

## Abstract

Flavivirus infection of cells induces massive rearrangements of the endoplasmic reticulum (ER) membrane to form viral replication organelles (ROs) which segregates viral RNA replication intermediates from the cytoplasmic RNA sensors. Among other viral nonstructural (NS) proteins, available evidence suggests for a prominent role of NS4B, an ER membrane protein with multiple transmembrane domains, in the formation of ROs and the evasion of the innate immune response. We previously reported a benzodiazepine compound, BDAA, which specifically inhibited yellow fever virus (YFV) replication in cultured cells and *in vivo* in hamsters, with resistant mutation mapped to P219 of NS4B protein. In the following mechanistic studies, we found that BDAA specifically enhances YFV induced inflammatory cytokine response in association with the induction of dramatic structural alteration of ROs and exposure of double-stranded RNA (dsRNA) in virus-infected cells. Interestingly, the BDAA-enhanced cytokine response in YFV-infected cells is attenuated in RIG-I or MAD5 knockout cells and completely abolished in MAVS knockout cells. However, BDAA inhibited YFV replication at a similar extent in the parent cells and cells deficient of RIG-I, MDA5 or MAVS. These results thus provided multiple lines of biological evidence to support a model that BDAA interaction with NS4B may impair the integrity of YFV ROs, which not only inhibits viral RNA replication, but also promotes the release of viral RNA from ROs, which consequentially activates RIG-I and MDA5. Although the innate immune enhancement activity of BDAA is not required for its antiviral activity in cultured cells, its dual antiviral mechanism is unique among all the reported antiviral agents thus far and warrants further investigation in animal models in future.

## Author summary

Emergence and re-emergence of yellow fever (YF) caused by the yellow fever virus (YFV) infection have posed a global public health threat in previously non-epidemic as well as endemic regions. The approximately 30% of mortality rate makes the outbreaks particularly devastating. In addition to the vaccination campaign and mosquito controls, antiviral drugs are important components in the toolbox for combating YF outbreaks. However, only two nucleotide analogue drugs developed for the treatment of other RNA virus infections are currently repurposed for the treatment of YF with uncertain clinical efficacy. BDAA is a benzodiazepine compound discovered as a potent YFV-specific antiviral agent in our laboratory. The work reported herein further demonstrates that BDAA interaction with the YFV NS4B protein may impair the integrity of viral RNA replication organelles, which not only inhibits viral RNA replication, but also results in the leakage of viral RNA into the cytoplasm to activate RIG-I-like RNA receptors and enhances the innate antiviral immune response. The unprecedented antiviral mechanism of BDAA highlights the essential role of the NS4B protein in viral RNA replication and the evasion of host cellular innate immunity.

## Introduction

Yellow fever (YF), a disease caused by infection of the yellow fever virus (YFV), was once considered as the most dangerous infectious disease with high fatality rate in the beginning of the 19th century. Subsequently, with the availability of a highly effective live-attenuated vaccine together with control of mosquito vectors, YF cases have been significantly reduced and the outbreaks have been mainly limited to the tropical and subtropical forested regions of Africa and South America. However, the re-emergence of YF outbreaks in previously low-risk regions such as Angola, Democratic Republic of Congo, and Brazil since 2016, indicates that actions must be taken to deal with the changing epidemiology of YF. Furthermore, the recent report of imported YFV-infected individual into Asia as well as the evidence that Asian-Pacific Aedes mosquitoes are competent vectors for YFV raise a concern for YF outbreaks in previously non-endemic and unvaccinated areas [1, 2]. Accordingly, new strategies in vaccination, therapeutics, and public health policies must be implemented to eliminate future global YFV threats.

The live-attenuated YF vaccine 17D, which was developed in 1936 by serial passages of the virus in chicken embryos, remains to be the best control measurement against YFV infection. However, the limitation in manufacture of the vaccine has caused a shortage in its supply in response to re-emerging outbreaks recently [3]. While new vaccines have been under development, combination of vaccination and therapeutic interventions should be paramount to prevent and manage the future YF outbreaks [4]. In a study performed during the 2018 outbreak of YF in Brazil, a 36% fatality rate was observed, and the high viral load was found to be a key determinant of disease severity, suggesting that an effective antiviral drug against YFV can be anticipated to significantly improve the clinical outcome of YF [5]. Antiviral therapy may also help control the outbreak by administration to potentially exposed individuals as a prophylaxis since the protection from vaccine will take weeks to become effective [6–8]. This approach is also important when YF vaccine is in short supply during unexpected outbreaks. Furthermore, for travelers to the endemic areas with potential risk of safely receiving YFV vaccine, prophylaxis antiviral drug can serve as an alternative prevention method [9]. However, the YF antiviral therapeutic candidates are currently limited to repurposed nucleoside analogs originally developed for hepatitis C virus (HCV) infection, among which BCX4430 (Galidesivir) [10] and Sofosbuvir [11] were found to inhibit YFV replication *in vitro* and *in vivo* in animal models. A phase 1 clinical trial to evaluate the safety, pharmacokinetics and antiviral effects in patients with YF or COVID-19 is currently ongoing *via* intravenous injection of BCX4430 (NCT03891420). Whereas, Sofosbuvir has been previously used to treat YFV-infected patients as compassionate use in two patients with acute liver failure [12]. Apparently, *bona fide* antiviral drugs against YFV are urgently needed for the control of YF outbreaks.

We recently reported the discovery of a benzodiazepine compound, BDAA, with potent antiviral effect against YFV specifically, but not other flaviviruses, in cell cultures and *in vivo via* oral dosing in hamsters. BDAA-resistant mutation had been mapped to proline 219 (P219) in the nonstructural 4B (NS4B) protein. The substitution of the NS4B P219 with serine confers YFV resistance to BDAA [13]. Furthermore, we have demonstrated that BDAA targets a post entry event and does not affect YFV polyprotein translation and processing [14] and there is a synergistic effect between BDAA and sofosbuvir in inhibiting YFV replication [15]. In the continuing efforts toward understanding the mode of action of BDAA, we found that in addition to inhibiting YFV replication, BDAA also specifically enhances the YFV-induced inflammatory cytokine response in infected cells in a RIG-I-like receptor (RLR)-dependent manner. To our knowledge, the dual antiviral and innate immune enhancing mechanisms are unprecedented and unique to BDAA, but not other NS4B-targeting antiviral agents against YFV and other flaviviruses.

## Results

### BDAA treatment enhances YFV-induced IFN-β expression at sub-optimal antiviral concentrations

**Fig 1:**
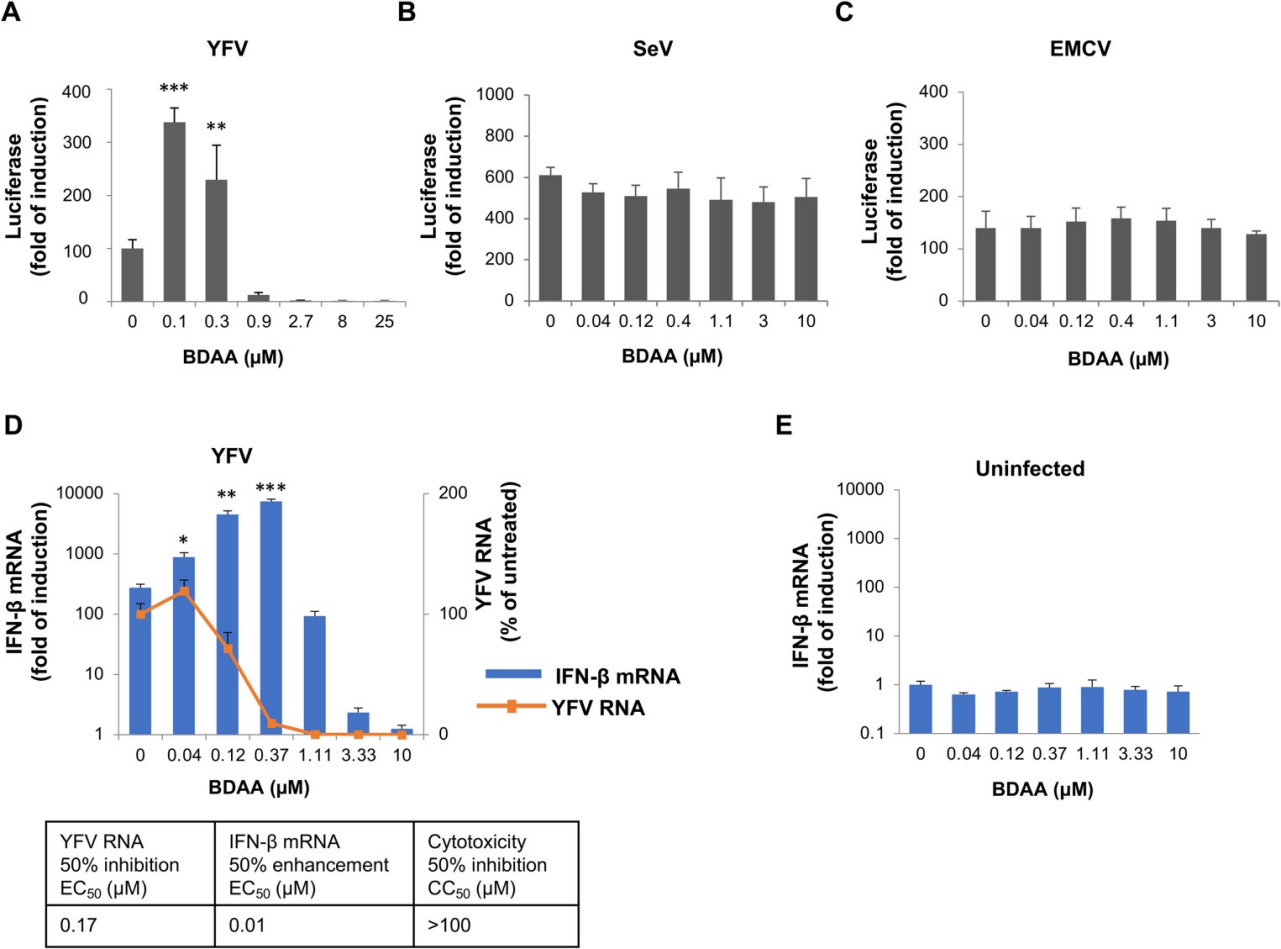
BDAA treatment specifically enhances YFV-induced IFN-β expression. **(A to C)** Effect on reporter expression in 293/IFNβLuc cells expressing luciferase under IFN-β promotor control. 293/IFNβLuc cells were seeded on 96-well plate and infected with YFV at MOI of 0.01 (A), Sendai virus (Sev) at 40 HAU (B), or encephalomyocarditis virus (EMCV) at MOI of 0.01 (C) for 1 h, followed by treatment with indicated concentration of BDAA. Luciferase activity was detected at 48 hpi and expressed as fold of induction relative to that in uninfected cells. Values represent average and standard deviation (STDV) from 3 independent experiments. **(D to E)** Effect on YFV RNA and IFN-β mRNA expression in 293/IFNβLuc cells. 293/IFNβLuc cells were either infected with YFV at MOI of 0.01 (D), or mock infected (E), for 1 h followed by treatment with indicated concentration of BDAA. Total cellular RNA was extracted at 48 hpi to detect YFV RNA and/or IFN-β mRNA by qRT-PCR. YFV RNA was expressed as percentage of YFV-infected and untreated control (D). IFN-β mRNA was expressed as fold of induction relative to that in uninfected cells (D) or untreated cells (E). BDAA concentrations for 50% inhibition of YFV RNA and 50% enhancement of IFN-β mRNA were calculated based on corresponding dose-responsive curves using GraphPad Prism 7. BDAA concentration for 50% inhibition of cell viability was determined by MTT assay (Sigma). Values represent average and standard deviation from 3 independent experiments. * indicates *P*<0.05, ** indicates *P*<0.01, ***indicates *P*<0.001 (IFN-β enhancement relative to no treatment control).

293/IFNβLuc is a HEK293-derived reporter cell line that expresses a firefly luciferase under the control of a human IFN-β promoter. Infection of this cell line with RNA viruses, including dengue virus, YFV, Sendai virus (SeV) and encephalomyocarditis virus (EMCV), activates the reporter gene expression that quantitatively correlates with the levels of virus replication and progeny virus production, and can be inhibited in a dose-dependent manner by known antiviral compounds [16]. Interestingly, during study of BDAA’s antiviral activity in this cell line, we fortuitously discovered that treatment of BDAA, starting at 1 h post infection (hpi) for 48 h, enhanced the reporter gene expression in cells infected with YFV, but not SeV or EMCV, at concentrations approximate to its EC_50_ value (Fig 1A–1C) [13]. This finding was further validated by demonstrating that treatment of YFV-infected, but not mock-infected 293/IFN-βLuc cells with BDAA using the same experimental schedule enhanced the IFN-β mRNA expression at the similar range of concentrations (Fig 1D and 1E). Moreover, treatment of YFV-infected HEK293 cells with BDAA or its three enantiomers enhanced IFN-β mRNA expression, exclusively at concentrations close to their respective EC_50_ values (S1 Fig). Interestingly, it appears that BDAA and its enantiomers specifically enhanced YFV-induced IFN-β response only at sub-optimal antiviral concentrations and as anticipated, near complete inhibition of viral RNA replication at higher, but non-cytotoxic, concentrations prevented the activation of IFN-β response (Figs 1D and S1). However, treatment of 293/IFNβLuc cells with YFV NS4B inhibitors CGG-4088 or CGG-3394 [17], NS5 polymerase inhibitor Sofosbuvir [11] or an inhibitor of host endoplasmic reticulum α-glucosidases (IHVR-19029) [18–20] inhibited viral RNA replication and IFN-β mRNA expression in a concentration-dependent manner, but none of these compounds enhanced IFN-β mRNA expression at any concentration tested (S2 Fig). These results clearly indicate that BDAA uniquely enhances IFN-β gene expression induced by YFV, but not other viruses, at sub-optimal antiviral concentrations under the defined treatment schedule.

### Both the inhibition of viral replication and the enhancement of IFN-β expression in YFV-infected cells by BDAA rely on its specific interaction with NS4B protein

**Fig 2:**
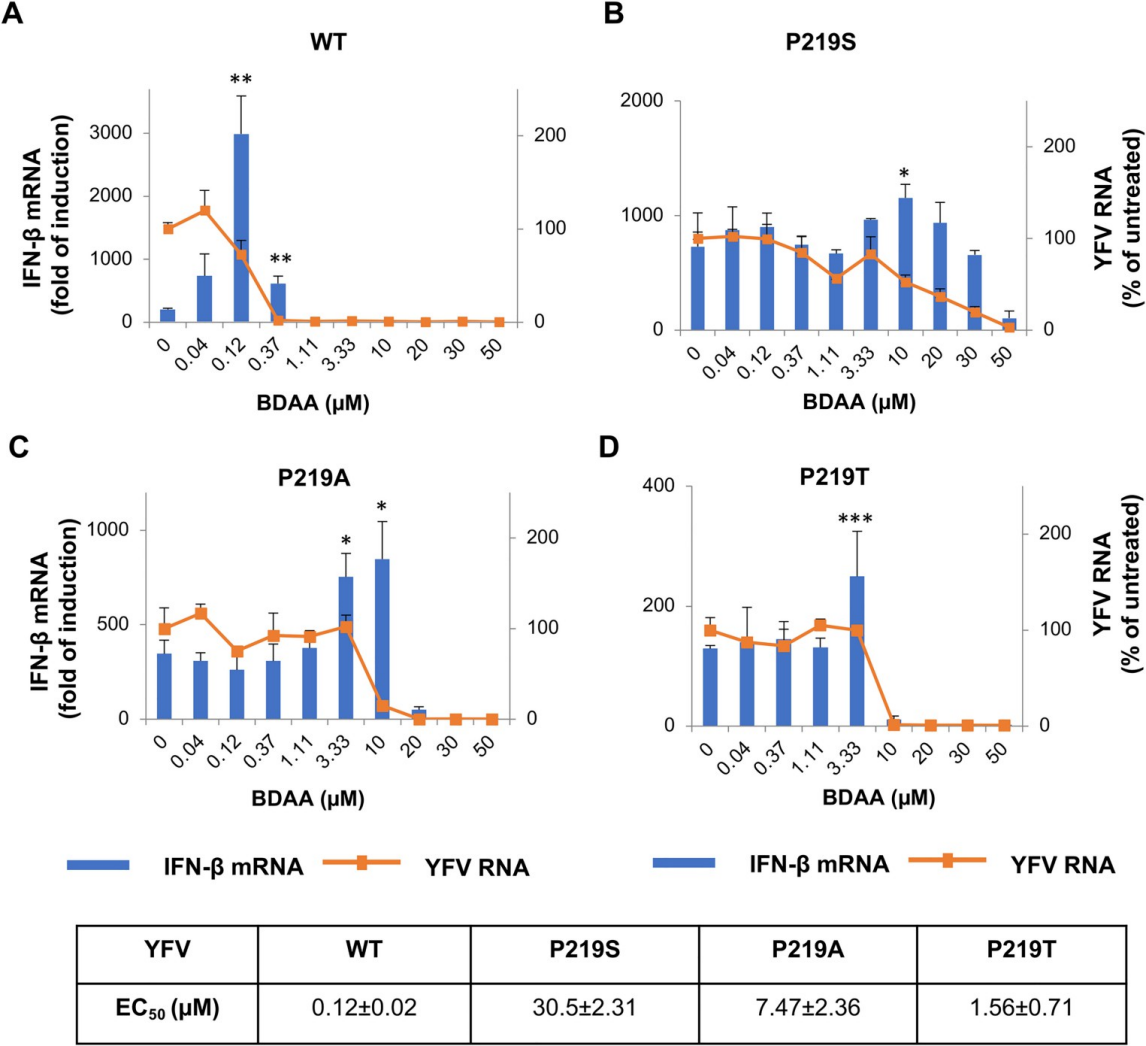
BDAA enhances IFN-β in cells infected with wild-type and BDAA resistant YFV at different concentrations. HEK293 cells were infected with wild-type YFV (**A**), or YFV NS4B P219S (**B**), P219A (**C**), P219T (**D**) mutant virus for 1 h, followed by treatment with indicated concentration of BDAA. Total cellular RNA was extracted at 48 hpi to detect YFV RNA and IFN-β mRNA by qRT-PCR. YFV RNA was expressed as percentage of untreated control. IFN-β mRNA was expressed as fold of induction relative to that in uninfected cells. Values represent average and standard deviation from 4 independent experiments. * indicates *P*<0.05, ** indicates *P*<0.01, ***indicates *P*<0.001 (IFN-β enhancement relative to no treatment control). EC_50_ values were calculated using GraphPad Prism 7.

We previously reported that the antiviral activity of BDAA depends on its specific interaction with YFV NS4B protein since the substitution of the NS4B residue P219 with several different amino acids conferred significant resistance to BDAA’s antiviral effect [13]. In order to determine whether BDAA enhancement of the IFN-β mRNA expression in YFV-infected cells also depends on its specific interaction with the NS4B protein, we compared the effects of BDAA treatment on the IFN-β expression in HEK293 cells infected with wild-type or BDAA-resistant YFVs. As shown in Fig 2, while BDAA enhanced the IFN-β mRNA expression in cells infected by all YFV strains, the concentrations required for significant enhancement of the cytokine response were always much higher in cells infected with BDAA-resistant YFVs, at suboptimal antiviral concentrations that are in accordance with the higher EC_50_ values of BDAA against the respective mutant YFVs. In marked contrast, treatment of YFV-infected HEK293 cells with CCG-4088, an anti-YFV compound with a resistant mutation mapped to residue K128 of NS4B transmembrane domain 3 (pTMD3) [17], dose-dependently inhibited YFV replication, but did not enhance IFN-β expression at any tested concentration (S2 Fig panel B). Hence, these results provide strong genetic evidence suggesting that both the inhibition of YFV replication and the enhancement of IFN-β expression by BDAA in YFV-infected cells require its specific interaction with NS4B at a structural motif including residue P219.

### BDAA enhanced a broad range of cytokine response in YFV-infected cells upon the onset of viral RNA replication

**Fig 3:**
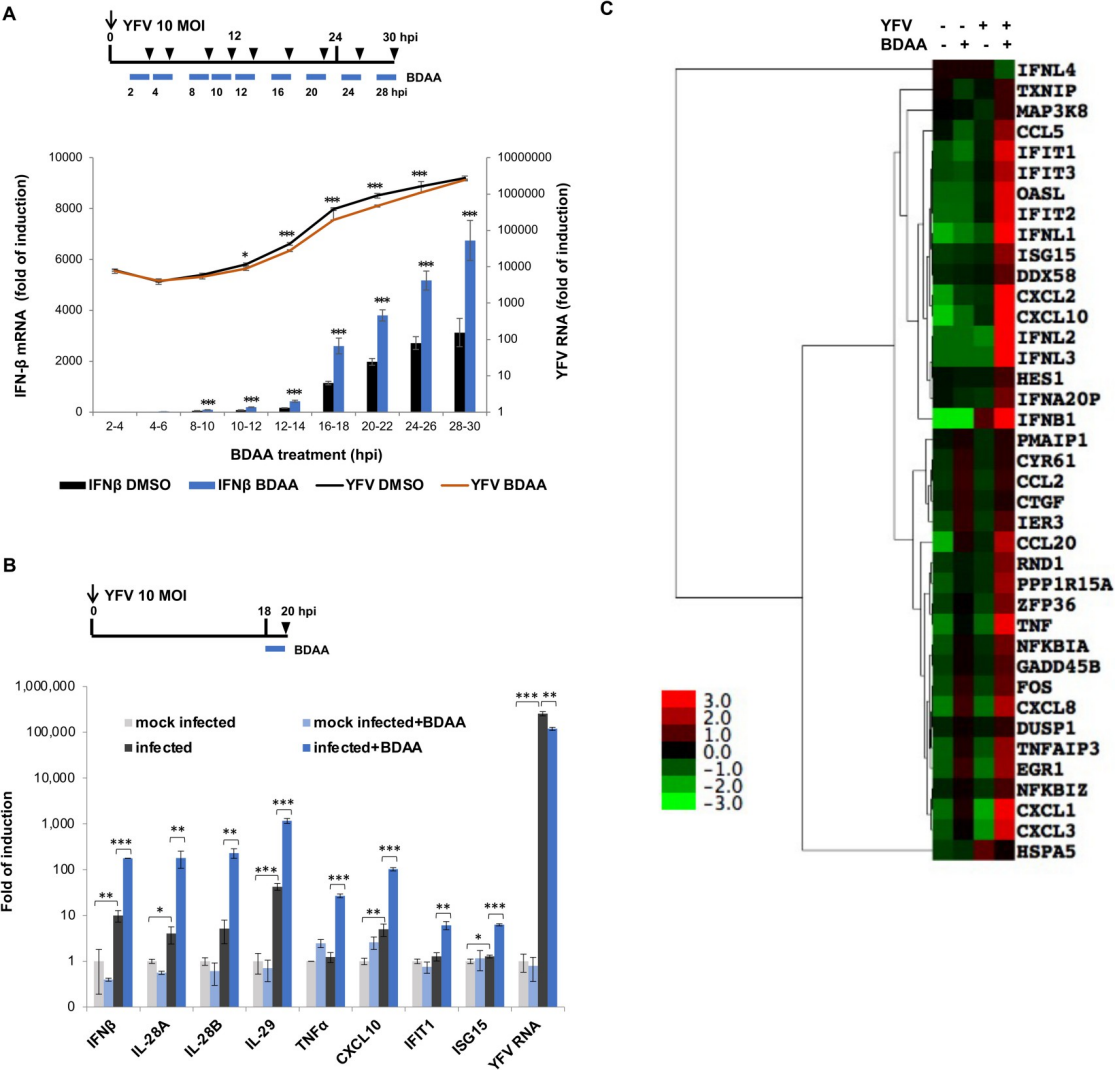
BDAA treatment enhances a broad range of YFV-induced cytokines, chemokines and ISGs. **(A)** Upper panel. Experimental design. HEK293 cells were infected with YFV at MOI of 10 for 1 h, followed by treatment with 3μM of BDAA for 2 h, starting at various time post infection. Total cellular RNA was extracted at the end of the 2 h treatment (indicated by arrow heads). Lower panel YFV RNA and IFN-β mRNA were detected by qRT-PCR and expressed as fold relative to that in uninfected cells. Values represent average and standard deviation from 4 independent experiments. * indicates *P*<0.05, ***indicates *P*<0.001 (IFN-β enhancement relative to DMSO mock treatment control). **(B)** HEK293 cells were either uninfected or infected with YFV at MOI of 10 for 1 h. At 18 hpi, cells were either mock treated with DMSO or treated with 3μM of BDAA for 2 h. Total cellular RNA was extracted at the end of the 2 h treatment (20 hpi). YFV RNA and indicated cytokine, chemokine or ISG mRNAs were detected by qRT-PCR and expressed as fold relative to that in uninfected cells. Numbers and bars represent average and standard deviation from 3 independent experiments. * indicates *P*<0.05, ** indicates *P*<0.01, ***indicates *P*<0.001. **(C)** Gene expression profile change across all samples was analyzed by RNAseq. The heat map listed up- or down-regulated genes with fold change greater than 1.5 and false discovery rate of less than 20%.

The results presented above indicate that BDAA significantly enhances YFV-induced IFN-β expression only at concentrations that partially inhibit viral RNA replication. More potent inhibition of viral replication at higher concentrations will profoundly reduce viral RNA, the ligand of cytoplasmic RNA sensors, and consequentially prevented YFV activation of IFN-β response. This interpretation of the results predicts that BDAA-enhanced IFN-β mRNA expression depends on the onset of viral RNA replication in infected cells. Therefore, we hypothesized that a short term BDAA treatment of YFV-infected cells to avoid significant reduction of YFV RNA may enhance the cytokine response in a broader range of BDAA concentration, including concentrations much higher than its EC_50_, only after the onset of viral RNA replication. Accordingly, HEK293 cells were infected with YFV at a MOI of 10 to ensure more than 99% of the cells were infected and then treated with BDAA, starting at the indicated time points post infection for a period of 2 h. As shown in Fig 3A, treatment of the YFV-infected HEK293 cells with 3 μM BDAA, starting at any time after the onset of viral RNA replication at 8 hpi, significantly enhanced IFN-β mRNA expression, along with significant yet modest inhibition of YFV replication. Specifically, less than 50% reductions in YFV RNA were observed over the 2 h treatment period with 3 μM BDAA initiated at any indicated time after the onset of viral RNA replication. These results thus indicate that BDAA rapidly enhances YFV-induced IFN-β expression upon the onset of viral RNA replication. As anticipated, we further demonstrated that BDAA treatment not only enhanced the expression of IFN-β, but also the expression of many other inflammatory cytokines, chemokines and interferon stimulated genes (ISGs) in HEK293 (Fig 3B) as well as HepG2 and Huh7 cells infected by YFV (S3 Fig). Moreover, an RNAseq analysis with a cutoff of false discovery rate of <0.2 and a fold change of >1.5 further demonstrated that the BDAA treatment of YFV-infected HEK293 cells at 18 hpi for 2 h increased the expression of a total of 39 cellular genes, 33 of which are inflammatory cytokines, chemokines or ISGs, and the remaining are mostly genes related to NFκB, TNF-α and MAPK signal transduction (Fig 3C and S1 Table). These results thus suggest that BDAA uniquely interacts with the YFV NS4B protein to trigger an enhanced activation of inflammatory cytokine responses.

### BDAA treatment induced dsRNA exposure in YFV-infected cells

The replication of flavivirus RNA genomes takes place in endoplasmic reticulum (ER) membrane-derived enclosed membranous webs/vesicles, *i*.*e*., replication complexes (RC). Specifically, the flavivirus nonstructural (NS) proteins NS2A, NS2B, NS4A and NS4B are integrated ER membrane proteins that drive extensive ER membrane re-arrangements for the formation of RC as well as other massive membrane alterations which are collectively referred as replication organelles (ROs) [21–23]. The RC accommodates viral genome replication so that the viral RNA replication intermediates are segregated from host innate immune sensors that recognize pathogen associated molecular patterns (PAMPs), such as viral RNA, in cytoplasm [24, 25]. It is well known that flavivirus NS4B proteins are multi-transmembrane proteins that play an essential role in the RC/RO formation [22, 26]. Particularly, the residue P219 is the amino acid immediately preceding the fifth transmembrane domain (pTMD5) of the YFV NS4B protein and may play an important role in the topogenesis of the NS4B protein and consequentially, the formation, integrity, and function of ROs [27, 28]. Accordingly, we hypothesized that interaction of BDAA with a NS4B structural motif including residue P219 may disrupt the integrity of YFV ROs, which not only inhibits viral RNA replication, but also results in the leakage of viral RNA replication intermediates, *e*.*g*., double stranded RNAs (dsRNAs), for the enhanced activation of cytosolic RNA sensors in YFV-infected cells.

**Fig 4:**
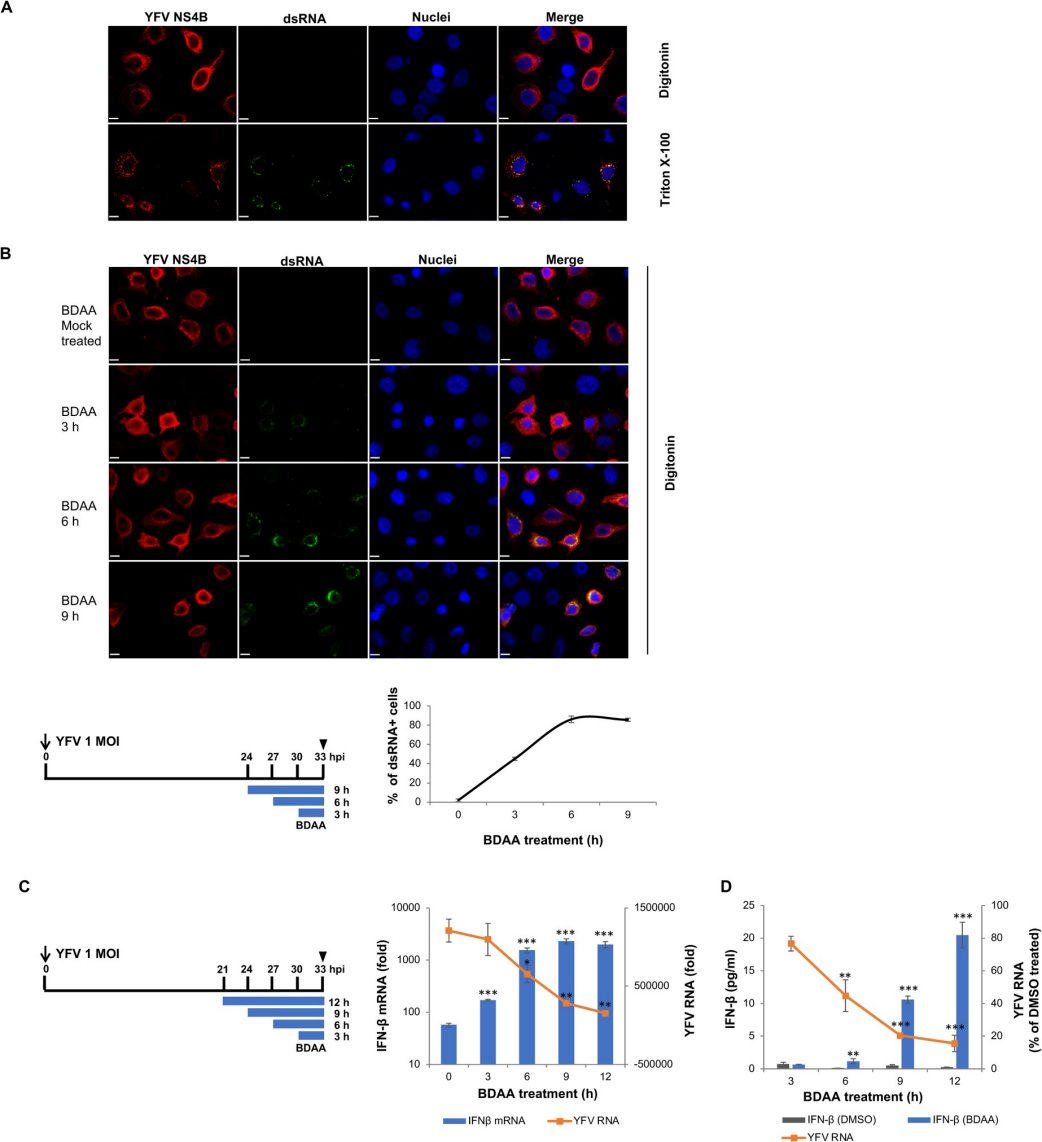
BDAA treatment induces the exposure of dsRNA from digitonin resistant YFV RO. (**A**) Huh7 cells seeded on coverslips in 24-well plate were infected with YFV at MOI of 1. Cells were fixed and permeabilized by digitonin (top row) or Triton X-100 (bottom row) at 24 hpi. YFV NS4B (red) and dsRNA (green) were visualized by immune fluorescence staining. Cell nuclei were stained by DAPI (blue). (**B**) Huh7 cells seeded on coverslips in 24-well plate were infected with YFV at MOI of 1 for 1 h, followed by treatment with DMSO or 5μM BDAA at indicated time post infection (as illustrated) for 3, 6, or 9 h. At 33 hpi, cells were fixed and permeabilized with digitonin. YFV NS4B (red) and dsRNA (green) were visualized by immunofluorescence staining. Scale bar is 100μm. Numbers of dsRNA positive cells were counted in three independent fields with at least 30 NS4B positive cells per field. Percentage of cells with dsRNA staining in YFV-infected cells (with NS4B staining) were calculated and expressed as average and STDV. **(C)** Huh7 cells seeded in 24 well plate were infected with YFV at MOI of 1 for 1 h followed by treatment with DMSO or 5μM of BDAA starting at indicted time for 3, 6, 9, or 12 h (as illustrated). Total cellular RNA was extracted at 33 hpi. YFV RNA and IFN-β mRNA were measured by qRT-PCR and expressed as fold relative to that in uninfected cells. Values represent average and standard deviation from 4 independent experiments. * indicates *P*<0.05, ** indicates *P*<0.01, ***indicates *P*<0.001 compared to no treatment controls. **(D)** Huh7 cells seeded in 96 well plate were infected with YFV at MOI of 1 for 1 h followed by treatment with DMSO or 5 μM of BDAA, starting at the indicted time for 3, 6, 9, or 12 h (as illustrated in panel C). Supernatants were harvested at 33 hpi to measure the secreted IFN-β. Values were determined after normalization to standard curve and express as average and standard deviation from 3 independent experiments. Total cellular RNA was extracted at 33 hpi. YFV RNA was measured by qRT-PCR and expressed as percentage of that in DMSO treated cells. Values represent average and standard deviation from 3 independent experiments. ** indicates *P*<0.01, ***indicates *P*<0.001.

To investigate this hypothesis, we first determined whether the BDAA treatment of YFV-infected cells induced dsRNA exposure in the cytoplasm. Treatment of mammalian cells with a mild detergent digitonin disrupts the plasma membrane, but not intracellular membranes. However, treatment with a stronger detergent, such as Triton X-100, efficiently disrupts both plasma and intracellular membranes. Interestingly, dsRNA could be detected by immunofluorescence staining with a monoclonal antibody only in YFV-infected (but not mock-infected) cells after permeabilization with Triton X-100, but not digitonin (Fig 4A). The dsRNA could only be detected in cells infected with YFV, as judged by the co-staining of dsRNA with YFV NS4B protein in the same cells. Furthermore, the dsRNAs visualized in YFV-infected cells were largely co-localized with not only YFV NS4B (Fig 4A) but also many other NS proteins including NS1, NS2B and NS3 (with the exception of NS5), as well as all three structural proteins (capsid, prM and envelope), as we reported recently [15]. These results are consistent with previous finding that dsRNA in YFV replication complex is protected within digitonin-resistant but Triton X-100-sensitive membrane structure [29, 30]. Interestingly, after initiation of viral replication, when the YFV-infected cells were treated with BDAA for 3, 6, or 9 h, dsRNAs became readily detectable in YFV-infected cells following digitonin permeabilization. The percentage of infected cells (NS4B positive cells) with dsRNA staining increased from less than 2% to 45% after BDAA treatment for 3 h and reached to greater than 86% with prolonged treatment (Fig 4B). This result indicated that BDAA treatment resulted in dsRNA exposure from digitonin resistant YFV ROs, which might be responsible for the enhanced activation of cytoplasmic RNA sensors. This interpretation is further supported by the finding that the magnitudes of BDAA-induced IFN-β mRNA enhancement in YFV-infected cells increased as the function of BDAA treatment duration and aligned perfectly with the extents of its induced dsRNA exposure (Fig 4C). YFV RNA level did not change dramatically after 3 h BDAA treatment, but significantly decreased after longer treatment durations (Fig 4C). Secreted IFN-β in culture media was similarly enhanced by BDAA treatment as a function of treatment duration (Fig 4D).

**Fig 5:**
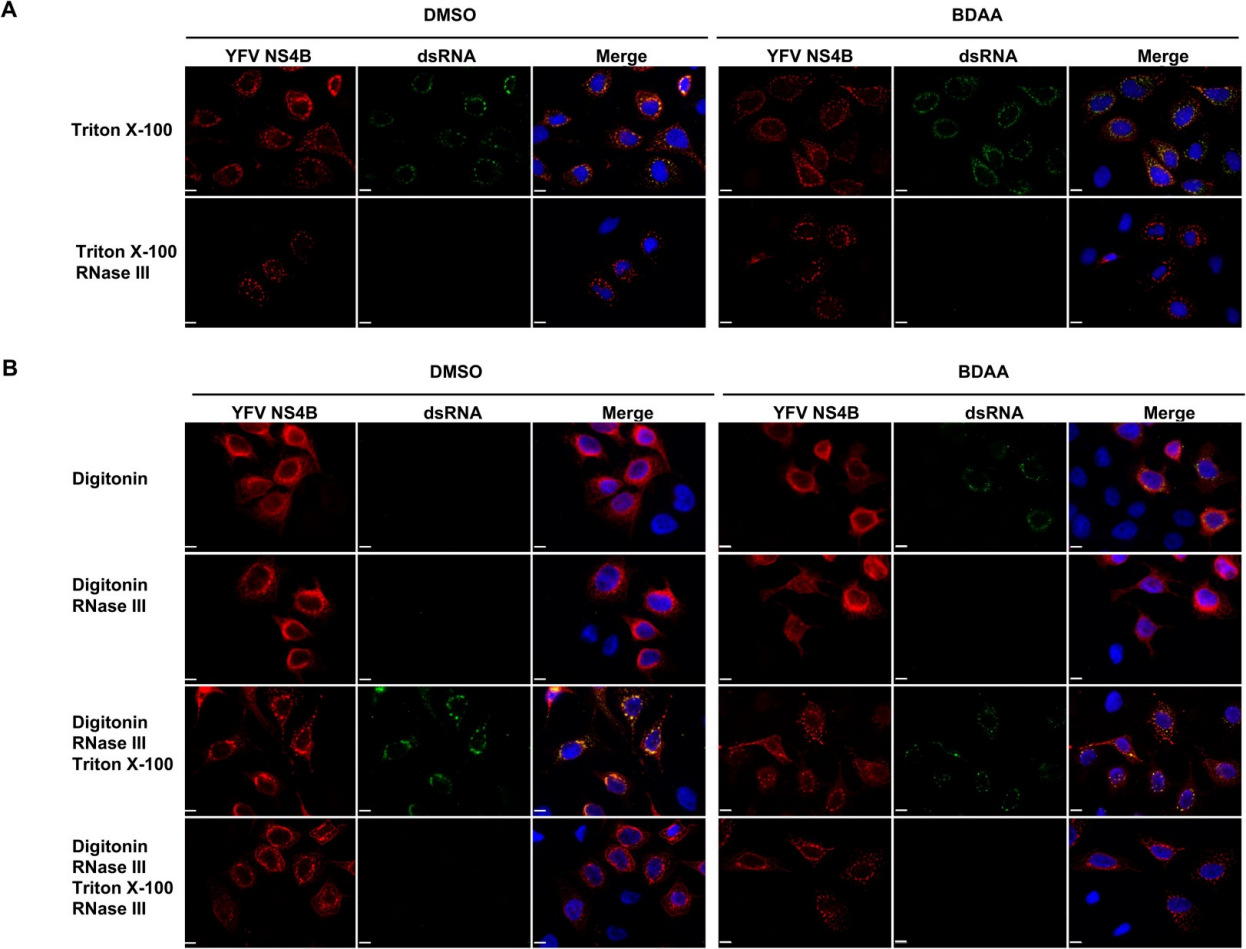
BDAA treatment induced exposure of dsRNA is sensitive to RNase III. Huh7 cells seeded on coverslips in 24 well plates were infected with YFV at MOI of 1 for 24 h followed by treatment with DMSO or 5μM BDAA for 6 h. **(A)** The cells were in situ permeabilized by Triton X-100 without or with RNase III treatment. **(B)** The cells were permeabilized by digitonin (top row). Alternatively, after in situ permeabilization with digitonin, cells were either additionally treated with RNase III (second row), RNase III treatment followed by permeabilization with Triton X-100 (third row), or RNase III treatment followed by permeabilization with Triton X-100 and another round of RNase III treatment (bottom row). Following the indicated treatment, the cells were fixed and followed by immunofluorescence staining using YFV NS4B or dsRNA antibodies. YFV NS4B (red), dsRNA (green) and cell nuclei (blue) were visualized. Scale bar is 100μm.

To further confirm the exposure of dsRNA induced by BDAA treatment, we tested its accessibility to the digestion by exogenous RNase III, a ribonuclease specific for dsRNA [31]. As anticipated, Triton X-100 treatment of YFV-infected Huh7 cells conferred the sensitivity of dsRNA to RNase III digestion (Fig 5A). However, after RNase III treatment of digitonin permeabilized YFV-infected cells, dsRNAs could still be visualized following Triton X-100 treatment (Fig 5B left panel, the third row). The Triton X-100 exposed dsRNA was again sensitive to RNase III digestion (Fig 5B left panel, the last row). Interestingly, upon treatment of YFV-infected cells with BDAA for 6 h, starting at 24 hpi, consistent with the result shown in Fig 4, not only large percentage of infected cells now had detectable dsRNAs following digitonin permeabilization, these dsRNAs were sensitive to RNase III digestion (Fig 5B right panel, the first and second rows), suggesting that the dsRNAs were exposed from the otherwise digitonin-resistant intact ROs upon several hours of BDAA treatment. Interestingly, following the digitonin permeabilization and RNase III treatment to digest the exposed dsRNA, when the BDAA-treated cells were further permeabilized with Triton X-100, RNase III sensitive-dsRNAs could still be detected in a significant portion of infected cells, *albeit* at a reduced fluorescent intensity, compared to that in DMSO treated cells (Fig 5B the third and the last rows). This later result suggests that not all ROs are sensitive to BDAA induced leakage. In another word, at least two structurally distinct ROs may exist in YFV-infected cells, which might represent newly formed and mature ROs, respectively. The above observations were made in not only Huh7 cells (Fig 5) but also HEK293 cells (S4 Fig).

**Fig 6:**
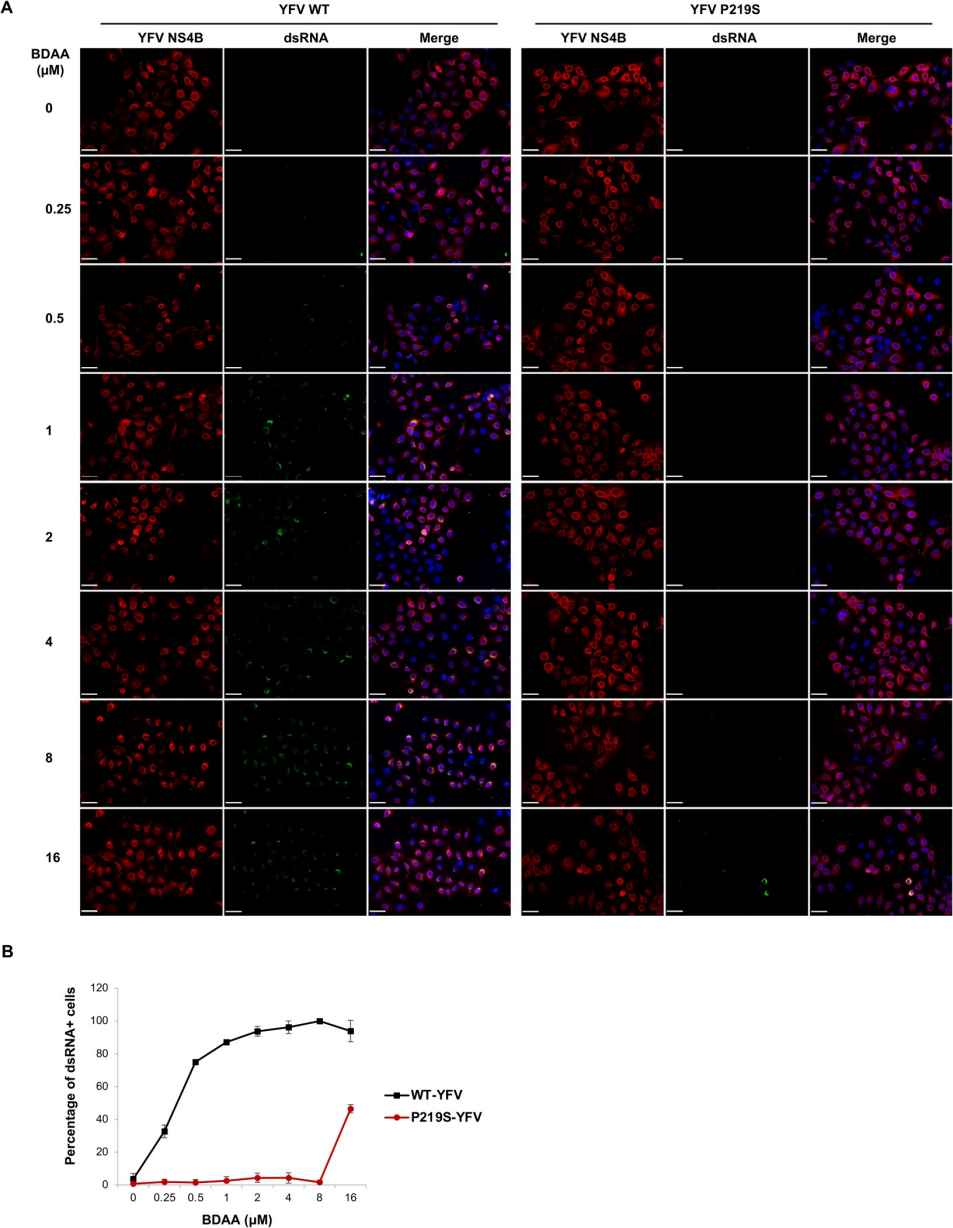
BDAA-induced dsRNA exposure in cells infected with WT YFV or BDAA-resistant YFV with P219S mutation. **(A)** Huh7 cells seeded in 24-well plate were infected with WT YFV or P219S YFV at MOI of 1. At 24 hpi, the cells were treated with DMSO or doses of BDAA for 6 h. The cells were then fixed and permeabilized with digitonin, followed by detection of YFV NS4B and dsRNA by immunofluorescent staining. NS4B (red), dsRNA (green) and cell nuclei (blue) were visualized. Scale bar is 50μm. (**B)** Numbers of dsRNA positive cells were counted in three independent fields with at least 30 NS4B positive cells per field. Percentage of cells with dsRNA staining in YFV-infected cells (with NS4B staining) were expressed as average and STDV.

Moreover, in agreement with the results presented in S2 Fig, the YFV NS4B inhibitor CCG-4088 that did not enhance YFV-induced IFN-β expression also failed to induce dsRNA exposure at comparably high antiviral concentrations (~10-fold of EC_50_) and treatment duration (S5 Fig). We further demonstrated that the exposure of dsRNA from digitonin-resistant ROs in cells infected by NS4B-P219S mutant YFV required much higher concentrations of BDAA (Fig 6). The dsRNA signals were also significantly weaker in cells infected with mutant virus after treatment with 16μM of BDAA, compared to cells infected with the wild-type virus and treated with 0.25μM of BDAA, under both conditions, approximately 40% of cells were dsRNA positive. These results reinforce the notion that the unique interaction of BDAA with NS4B at the structural motif including residue P219 specifically induces the exposure of dsRNA from YFV ROs.

### Both RIG-I and MDA5 play a role in YFV activation of inflammatory cytokine response and are enhanced by BDAA treatment in HEK293 cells

RIG-I like receptors (RLRs), including retinoic-acid-inducible protein I (RIG-I) and melanoma differentiation antigen 5 (MDA5), are the primary viral RNA sensors for the activation of the innate immune response in flavivirus-infected cells [32, 33]. Particularly, the essential and nonredundant roles of RIG-I and MDA5 in the detection and control of West Nile virus infection by recognizing 5’ triphosphate and double-stranded RNAs, respectively, have been well documented [34, 35]. Both RIG-I and MDA5 are involved in responses to dengue virus serotype-2 (DENV-2) infection in mouse embryonic fibroblasts [36] and Zika virus infection in human trophoblasts [37]. Concerning the role of RLRs in YFV infection, it was reported that replication of YFV vaccine strain YF-17D in human pDCs and pDC-like cell lines, and YFV reference strain *Asibi* in human hepatoma cells stimulated type I IFN production through activation of RIG-I [38, 39].

**Fig 7:**
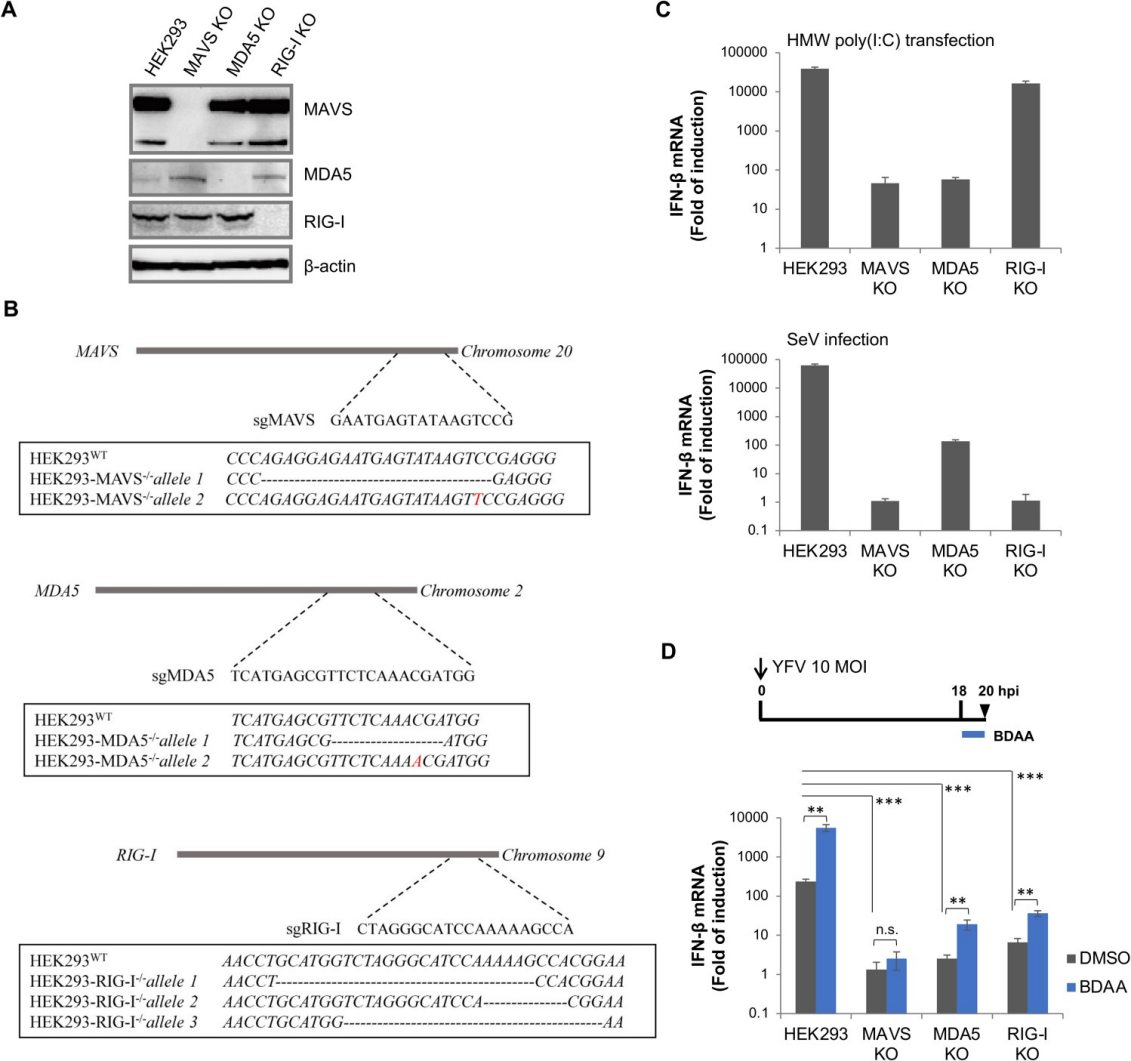
Characterization of HEK293 cells with RIG-I, MDA5 or MAVS knockout. **(A)** Expression of RIG-I, MDA5 or MAVS in wildtype and knockout cell lines were detected by western blot assay, using β-actin as a loading control. **(B)** Analysis of sequence flanking the corresponding sgRNA targeting site in RIG-I, MDA5 or MAVS KO cell lines. Dotted line represents base deletion and red letter represents base insertion. **(C)** Functional analysis of KO cell lines. Parental HEK293 or derived cell lines with indicated gene KO were seeded in 24-well plate and either infected with SeV or transfected with high molecular weight (HMW) poly (I:C). Total RNA was extracted at 24 h later and IFN-β mRNA was measured by qRT-PCR and expressed as fold of induction (average ± STDV, n = 3). **(D)** Parental HEK293 or derived cell lines with indicated gene KO were seeded in 24-well plate and infected with YFV at MOI of 10. At 18 hpi, the cells were treated with DMSO or 3μM of BDAA for 2h. Total RNA was extracted at 20 hpi. IFN-β mRNA was measured by qRT-PCR and expressed as fold of induction (average ± STDV, n = 3). n.s. indicates not significant, ** indicates *P*<0.01, ***indicates *P*<0.001 compared to DMSO mock treatment controls.

To determine the roles of RLR pathway in YFV induction and BDAA enhancement of cytokine response, we created HEK293-derived cell lines deficient in the expression of RIG-I, MDA5 or MAVS, the central adaptor for RLR signaling, by CRISPR/Cas9 gene editing technology. The knockout of specific gene expression in each cell line was confirmed by Western blot assays (Fig 7A) and sequencing analyses of the chromosomal DNA at the respective guide RNA binding sites (Fig 7B). The functionality of RLR signaling in parental HEK293 and the three knockout cell lines were also characterized. As shown in Fig 7C, consistent with previous reports, while the induction of IFN-β mRNA expression by high molecular weight poly(I:C) transfection was almost completely abolished in MDA5 knockout cells, SeV-induced IFN-β expression was more significantly compromised in RIG-I knockout cells [33, 40]. Also as expected, MAVS knockout profoundly abolished IFN-β induction by both types of the RNA ligands (Fig 7C). These results indicate that all the knockout cells with deficiency in RLR signaling displayed the expected phenotypes in responding to well-characterized RLR ligands. To determine the role of each of the three genes in YFV replication and induction of inflammatory cytokine response, parental HEK293 and each of the three knockout cell lines were infected with YFV and infected cells were harvested at the indicated time points post infection for qRT-PCR quantification of intracellular viral RNA and IFN-β mRNA (S6 Fig). The levels of YFV RNA in each of the RLR pathway component knockout cell lines were only slightly higher (less than 6-fold) than that in parental HEK293 cells (S6 Fig, panel A). However, induction of IFN-β mRNA expression was drastically reduced in both RIG-I and MDA5 knockout cells and even more profoundly reduced in MAVS knockout cells up to 36 to 48 h post infection. However, after 48 h post infection, IFN-β mRNA was significantly induced in all the three knockout cell lines infected by YFV (S6 Fig, panel B). Because the later surge of IFN-β mRNA induction occurred not only in RIG-I or MDA5 knockout cells, but also in MAVS knockout cells, the results strongly indicate the activation of innate immune pathways other than RLRs in the later stage of YFV infection in these cell lines. It is rather interesting that the robust activation of IFN response in HEK293 cells only modestly inhibited YFV replication at the condition of a low (0.1) MOI infection. In fact, similar observations that RLR silencing did not significantly affect viral replication in cell cultures had also been reported for other flaviviruses as reviewed by Valadão *et al*. in 2016 [41].

Finally, we examined the pathways responsible for the BDAA enhanced IFN-β production in YFV-infected cells. As shown in Fig 7D (blue bars), in both RIG-I and MDA5 knockout cells infected with YFV, BDAA treatment still enhanced IFN-β mRNA production. However, both the YFV-induced and BDAA-enhanced IFN-β production were almost completely abolished in MAVS knockout cells. These results clearly indicate that at least in the early time post YFV infection (18 hpi), both RIG-I and MDA5 play important roles in the induction of inflammatory cytokine response and BDAA treatment enhanced the cytokine response by both RNA sensors. These results imply that the RNA ligands responsible for the YFV-induced innate immune response may share the same molecular features with those responsible for BDAA enhanced cytokine responses, presumably both are various types of viral RNA, including dsRNA, derived from viral replication complexes.

### Morphology and structural alterations induced by BDAA treatment in YFV-infected cells

**Fig 8:**
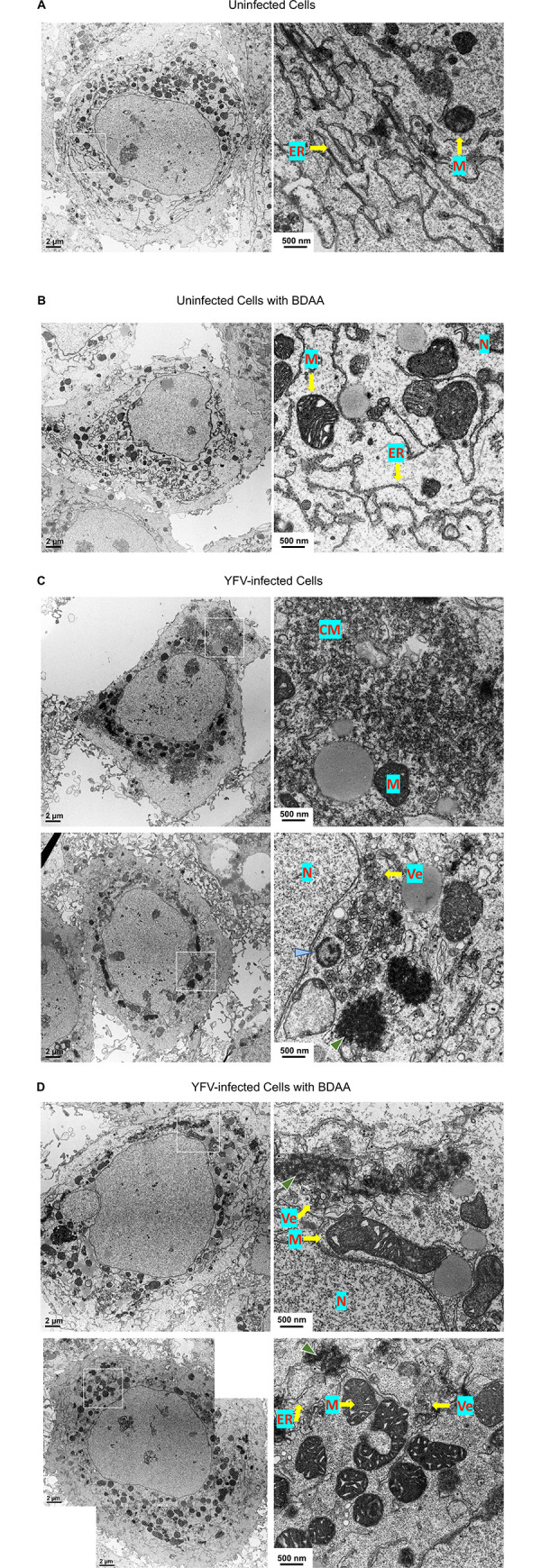
Morphology of YFV-infected Huh7 cells. Huh7 cells were seeded on Costar 3801-Clear polyester membrane. Uninfected cells mock treated (A) or treated with BDAA (B) served as controls. For cells infected with YFV at MOI of 10, at 24 h post infection, the cells were either mock treated (C), or treated with 3 μM of BDAA (D) for 6 h. Cells were fixed, sectioned, and imaged by transmission electron microscopy. The areas boxed in the panels on the left are shown at higher magnification on the right. Scale bars in each panel are as indicated. N, nucleus; M, mitochondria; CM, convoluted membrane; Ve, virus-induced vesicle; green arrowhead, high-density nucleic acid staining; blue arrowhead, horseshoe-shaped structure.

To further investigate the mechanism by which BDAA treatment induces dsRNA exposure and RIG-I/MDA5 activation, we attempted to visualize the structural alterations of YFV ROs induced by BDAA treatment *via* transmission electron microscope. As shown in Fig 8, uninfected Huh7 cells, in the absence or presence of BDAA treatment, showed typical hepatoma cell morphology, including the endoplasmic reticulum (ER). Similar to cells infected by other flaviviruses [24, 42], Huh7 cells infected with YFV showed ROs with convoluted membranes (CM), virus-induced vesicles (Ve) and high-density nucleic acid staining including a horseshoe-shaped structure typical of the site of viral replication. However, BDAA treatment reduced the area and density of ROs, such as CM, cytoplasmic nucleic acid staining and Ve in YFV-infected cells, suggesting that there is less replication and/or less compacted RNA. While these results clearly indicate that the short-term treatment of BDAA induced dramatic structural alterations of YFV ROs, the precise structure basis underlines the exposure of viral RNA replication intermediates from the ROs remains to be revealed. Apparently, more detailed examination of the intracellular ultrastructures in YFV infected cells using advanced EM technologies, such as 3D electron tomography (ET), immunoEM, correlative light and electron microscope (CLEM) or cryoEM, in future are required to establish the relationship between specific structure alterations of YFV ROs and the release of viral RNA replication intermediates.

## Discussion

**Fig 9:**
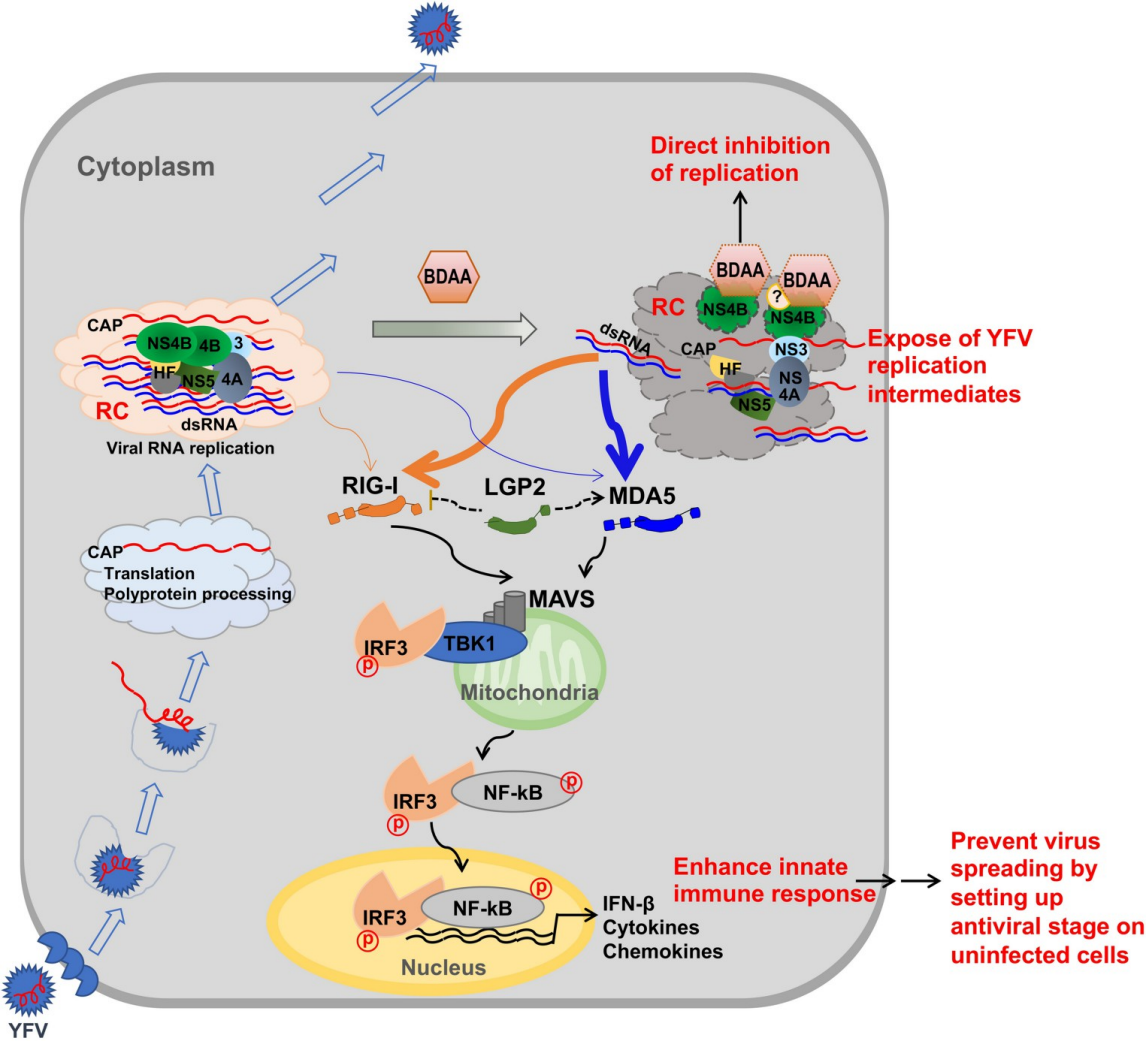
Illustration of the mode of BDAA action. See text for detailed discussion.

BDAA is a small molecular antiviral agent against YFV with drug resistant mutation mapped to P219 of NS4B protein [13]. We demonstrated in this study that BDAA treatment of YFV-infected cells not only inhibited viral replication, but also enhanced the YFV-induced inflammatory cytokine response in a manner depending on viral RNA replication (Figs 1–3). The BDAA enhancement of the innate immune response in YFV-infected cells is qualitatively and quantitatively co-related to the exposure of dsRNA in the cytoplasm of infected cells upon BDAA treatment (Figs 4 and 5). Further analyses revealed that BDAA treatment enhanced viral RNA-induced cytokine response *via* the activation of both RIG-I and MDA5 (Fig 7). Meanwhile, two independent lines of evidence also support the notion that the BDAA inhibition of viral replication is independent of its enhanced activation of the innate immune response in virally infected cells. First, although treatment of YFV-infected cells immediately after inoculation with sub-optimal concentrations of BDAA enhanced innate immune response, treatment with higher concentrations of BDAA inhibited both YFV replication and IFN-β response (Figs 1 and 2). Second, BDAA inhibited YFV RNA replication at a similar efficacy in parental HEK293 cells as well as MAVS, MDA5 or RIG-I knockout cells (S7 Fig) with comparable EC_50_ and EC_90_ values when the cells were infected with either low or high MOI (S2 Table). Importantly, NS4B P219 mutations conferred resistance to both BDAA inhibition of viral replication and the enhancement of dsRNA exposure and cytokine response to a similar extent (Figs 2 and 6). These findings together strongly support a model in which the binding of BDAA to the NS4B protein at a structural motif including residue P219 not only inhibits viral RNA replication, but also induces the exposure of viral RNA replication intermediates, *e*.*g*., dsRNAs, for enhanced RIG-I and MDA5 activation (Fig 9). In support of the dual activities of BDAA on viral replication and enhanced innate immune activation, we further demonstrated that BDAA treatment induced drastic alteration of YFV ROs (Fig 8). To our knowledge, this “one stone, two birds” pharmacological mechanism of BDAA is unprecedented among previously reported antiviral agents.

The formation of flavivirus ROs is known to be driven by the interactions between viral nonstructural proteins as well as with host cellular factors [26, 43–45]. The dependence of the dual pharmacological effects of BDAA on the P219 residue in the YFV NS4B protein implies that the binding of BDAA to the NS4B at a structure motif including residue P219 triggers the structural alterations of ROs membrane leading to both inhibition of viral RNA replication and release of viral RNA replication intermediates for enhanced activation of the RNA sensors. Concerning the unique role of NS4B in YFV evasion of innate immune response, it is very interesting to point out that the baseline IFN-β expression in P219S mutant virus infected cells is higher than that in wild-type virus infected cells, suggesting that the mutation in NS4B protein may result in altered ROs structure/integrity and reduced ability to evade the recognition by the cytoplasmic RNA sensors (Fig 2). This hypothesis will be further examined in future EM study.

Flavivirus NS4B proteins are multi-transmembrane ER proteins that have similar membrane topologies [26]. Several structurally distinct compounds have been identified to inhibit the replication of DENV, Japanese Encephalitis virus (JEV) or YFV through targeting the NS4B [17, 46–51]. While the drug-resistant mutations to other NS4B inhibitors are primarily mapped to the transmembrane domains, the BDAA-resistant mutation, P219, is located at the lumen side of the ER immediately before the fifth transmembrane domain (TM5). Interestingly, it was reported that the drug-resistant mutations to NITD-688, a pan-serotype DENV specific NS4B inhibitor, were mapped to T195, T215 and A222 of NS4B. Furthermore, drug-resistant mutations to another pan-serotype DENV specific NS4B inhibitor, JNJ-A07, were mapped to V91, L94 and T216 [52]. While V91 and L94 are located in the ER lumen loop between the second (TM2) and the third (TM3) transmembrane domains, and A222 is located within TM5, residues T195, T215 and T216 are all located at the loop in the ER lumen between the fourth (TM4) and fifth (TM5) transmembrane domains [53]. Especially, T215 and T216 residues in the DENV NS4B correspond to the G218 and P219 in YFV NS4B, suggesting that the area immediately preceding TM5 might be an important target site of flavivirus NS4B inhibitors. It is also worth noting that many other drug-resistant mutations in flavivirus NS4B confer cross resistance to multiple structurally unrelated inhibitors, suggesting that those amino acid residues are hot spots of intramembrane protein-protein interaction important for the assembly and/or function of ROs [13, 22]. The unique location of BDAA-resistant residue and the dual topology of the flavivirus NS4B TM5 in the ER membrane before and after polyprotein processing at NS4B/NS5 junction [13, 27] provide initial insights for further investigation of the mechanism of BDAA interaction with NS4B and induction of RO structural alterations, which are essential to uncover the molecular mechanism of its dual pharmacological effects. Considering that NITD-688 and JNJ-A07 may bind to a similar region of the DENV NS4B, it will be very interesting to investigate whether these compounds also enhance the innate immune response in DENV-infected cells. In agreement with the reported role of RIG-I and MDA5 in the flavivirus induction of the innate immune response in infected cells, our results clearly demonstrated that both RIG-I and MDA5 play an important role in the YFV-induced inflammatory cytokine response in HEK293 cells (S6 Fig). Interestingly, knockout of MAVS completely prevented IFN-β mRNA induction within 48 h of YFV infection. However, significant amounts of IFN-β mRNA induction also occurred in MAVS knockout cells after 48 hpi (S6 Fig). This finding indicates that while RIG-I and MDA5 are solely responsible for the activation of IFN-β response in the early phase of YFV infection, additional pathway(s) are activated to induce IFN-β expression in the later phase of the YFV infection. While it is possible that the later surge of MAVS-independent IFN-β induction is due to the activation of the cGAS-STING pathway by the release of mitochondrial and/or nuclear DNA upon YFV-induced cellular stress and structural damages in the later phase of the infection [13, 54, 55], the pathobiological significance of this MAVS-independent cytokine response deserves further investigation in infected animals.

Studies in gene knockout mice indicate that RLR and type I IFN pathways play an important role in the control of flavivirus infection *in vivo*. For instance, Zika virus (ZIKV), DENV and YFV infection of mice lacking one or two type I IFN- receptors or STAT2 result in systemic and fatal diseases [56–59]. In addition to the induction of IFN and inflammatory cytokine-mediated antiviral immune response, the optimal priming of adaptive immunity against flavivirus infection also depends on the activation of RIG-I and/or MDA5 by viral RNA [60, 61]. For instance, knockout of RIG-I or MDA5 each reduced innate immune response and increased mortality of West Nile virus (WNV) infection in mice. WNV infection of RIG-I and MDA5 double-knockout mice or MAVS knockout mice failed to induce innate immune response and scrubbed to the viral infection [34]. In addition to the enhanced viral replication and dissemination with early viral entry into the central nerve system, WNV infected MAVS knockout mice demonstrated uncontrolled inflammatory cytokine/chemokine response due to the lack of the expansion of regulatory T cells and lack of neutralization antibody production [62, 63]. Although deficiency of RIG-I, MDA5 or MAVS in HEK293 cells only modestly increased the levels of YFV RNA (S6 Fig), the RLR pathway has been shown to play a critical role in the control of flavivirus infection and immunopathogenesis in vivo. Thus, we believe that in addition to its potent direct-acting antiviral activity, BDAA treatment of YFV infection may also play a role in shaping the host antiviral innate and adaptive immune response in vivo *via* enhanced activation of RIG-I and MDA5 RNA sensors.

## Materials and methods

### Cell lines

HEK293 (ATCC CRL-1537) and HepG2 (ATCC HB-8065) were purchased from ATCC. Huh7 cells were obtained from Dr. Charles M. Rice at Rockefeller University and cultured in DMEM, 10% fetal bovine serum (FBS), 1X non-essential amino acid (NEAA) [64]. The 293/IFNβLuc cell line was established based on HEK293 with reconstitution of an interferon β promotor driven luciferase as previously described [16, 65].

#### Establishment of gene-knockout cell lines using the CRISPR/Cas9 system

HEK293 cell lines with MAVS, MDA5 or RIG-I gene knockout, were established using abm CRISPR All-in-One set (abm, Cat no: C442, K1017405, K0575405, 402171110595). The single guide RNA (sgRNA) sequence for each of the target genes are as shown below. MAVS: GAATGAGTATAAGTCCG, MDA5: TCATGAGCGTTCTCAAACGA, RIG-I: CTAGGGCATCCAAAAAGCCA. Plasmid encoding sgRNA or Cas9 was transfected into 293T Lenti-X cells by using the Lenti-X Packaging Single Shots (TAKARA, Cat no: 631276) according to the manufacture’s protocol. Supernatant containing the lentiviral particles was harvested at 72 h post transfection and used for transduction of the target cells. Single cell clones were selected by culturing the transduced cells in medium containing 0.6 μg/ml puromycin (Gibco). The Knockout of target gene expression was validated by Western blot assay and DNA sequencing verification of targeted genes.

### Viruses

Yellow fever virus was generated from a cDNA clone, pACNR/FLYF-17Dx, a gift from Charles Rice at Rockefeller University [13, 66, 67]. YFV cDNA clones with mutation at NS4B P219 (YFV P219S, P219A and P219T) were generated based on pACNR/FLYF-17Dx, as described previously [13]. Sendai virus (SeV) (strain 52, ATCC VR-105) and encephalomyocarditis virus (EMCV) (ATCC VR-1762) were purchased from ATCC.

### Chemicals

BDAA [chemical name: 2-((S)-3-((S)-sec-butyl)-7-chloro-2-oxo-5-phenyl-2,3-dihydro-1H-benzo[e][1, 4]diazepin-1-yl)acetic acid] was synthesized by SRI through the NIAID preclinical service program. CGG-4088 and CGG-3394 were purchased from ChemBridge. Sofosbuvir. IHVR-19029 and BDAA isomers were synthesized and purified in house with >95% purity. High molecular weight (HMW) poly I:C was purchased from Invivogen.

### Luciferase assay

293/IFNβLuc cells were cultured in 96-well black wall plates overnight and infected with YFV at a MOI of 0.1. At 48 h post-infection, luciferase activity was measured using the Steady-Glo Luciferase Assay System (Promega) [16].

### Real time quantitative reverse transcription PCR assay

Total cellular RNA was extracted using TRIzol (ThermoFisher Scientific). The relative amounts of viral RNA and β-actin mRNA were determined with a quantitative one-step reverse transcription-PCR (qRT-PCR) assay using the LightCycler 480II device (Roche). The sequences of primers used in this study are listed in S3 Table with β-Actin serving as the internal control.

### RNAseq analysis

HEK293 cells were either uninfected or infected with YFV at s MOI of 10 for 1 h. At 18 hours post infection (hpi), the cells were either mock treated with DMSO or treated with 3μM of BDAA for 2 h. Total cellular RNA was extracted by using TRIzol reagent (Invitrogen). Poly (A) RNA were purified and subjected for sequencing analyses using Illumina Hiseq2500 at the Genomics Facility of Fox Chase Cancer Center. Tophat algorithm [68] was implemented to align raw sequence reads to human genome (HG38). Transcript assembly and abundance estimation were done with Cufflinks [69], followed by using Cuffdiff [70] to assess differential expression, as we described previously [71]. Multiple testing was adjusted with default method implemented in Cuffdiff. Genes with false discovery rate (FDR) <0.2 and fold change (FC) >1.5 were considered as significant.

### In situ permeabilization and Immunofluorescence assay

Huh7 or HEK293 cells were seeded on round coverslips (Neuvitro Corporation) in 24-well plate and infected with YFV. To perform the in situ permeabilization of the cellular membrane, the cells were washed with ice cold buffer C (20mM HEPES, PH7.7, 110mM potassium acetate, 2mM magnesium acetate and 1mM EDTA) and then treated with buffer C containing either 50 μg/ml of digitonin or 1% Triton X-100 (Promega) for 5 min on ice. Alternatively, following permeabilization with digitonin, the cells on the coverslips were further treated with buffer C containing 1% Triton X-100 for 5 min on ice. For dsRNA digestion, cells were incubated with RNase III (1 U/μl) (ThermoFisher Scientific) for 30min at room temperature after permeabilization with indicated method. The cells were then fixed with 3.5% paraformaldehyde (PFA) for 15 min followed by immunofluorescence staining. For regular immunofluorescence staining, the cells were fix first with 3/5% PFA followed by permeabilization with 50 μg/ml digitonin or 1% Triton X-100 as we describe previously [15]. Briefly, the cells were incubated with primary antibodies against dsRNA (J2, English & Scientific Consulting, Cat. No. MABE1134) or YFV NS4B (GeneTex, GTX134030) at 4°C overnight. Subsequently, the cells were washed three times with 0.1% Tween-20 in PBS (PBST) and stained with Alexa Fluor 488-conjugated anti-mouse and 594-conjugated anti-rabbit secondary antibodies and cell nuclei was counterstained with 4’,6-diamidine-2’-phenylindole dihydrochloride (DAPI). Finally, the coverslips were accreted on glass slides by ProLong Gold Antifade Mountant (ThermoFisher Scientific). Images were sequentially acquired on an FV1000 confocal microscope (Olympus) with a PlanApoN 60×/1.42 numerical aperture objective (Olympus).

### ELISA assay to detect secreted IFN-β

Cell culture media were collected from cells and the IFN-β was measured using an ELISA kit (DIFNB0, R&D Systems) following the manufacturer’s protocol.

### Western blot assay

Cells were lysed with 1× NuPAGE LDS Sample Buffer (Invitrogen) containing 2.5% β-mercaptoethanol. Cell lysates were loaded on a NuPAGE Novex 4–12% Bis-Tris gel (Invitrogen) and following electrophoresis, transferred onto a nitrocellulose membrane (Invitrogen). The membranes were then probed with an antibody against MAVS (CellSignal 3993S), MDA5 (Cell Signal 5321S) or RIG-I (CellSignal 3743S). The bound antibodies were visualized with IRDye secondary antibodies (IRDye 800CW Goat anti-Rabbit IgG or IRDye 680RD Goat anti-Mouse IgG) and imaged with the LI-COR Odyssey system. β-actin served as the loading control.

### High-content imaging assay

Examination of antiviral activity using high-content imaging assay was essentially carried out as previously described [15]. Briefly, cells were cultured in 384-well plates overnight and infected with YFV as described in the legend of S7 Fig. At 48 h post-infection, cells were fixed with 4% PFA and probed with a rabbit polyclonal antibody against YFV NS4B (GTX134030) and visualized with Goat anti-Rabbit 594 IgG (H+L) (ThermoFisher A-11012). Cell nuclei were stained with DAPI. Six fields per well in 384-well plate were analyzed using CellInsight CX7 High-Content Screening Platform (ThermoFisher).

### Ultrathin sectioning and transmission electron microscopy

Huh7 cells were grown on Costar 3801-Clear polyester membrane with 0.4μm pore size (Corning). Confluent cells were washed with 0.1M sodium cacodylate (pH 7.3) buffer and fixed twice with 2.5% glutaraldehyde (Electron Microscopy Sciences) in 0.1M cacodylate buffer at room temperature for 1 h, followed by washing with 0.1M sodium cacodylate buffer for three times. Plastic embedding and ultrathin sectioning were performed in the electron microscopy core facility at the Cleveland Center for Membrane & Structural Biology, Case Western Reserve University. Stained ultrathin sections were observed in a Tecnai 12 electron microscope and images were recorded with a CCD camera.

### Statistical analysis

*P* values were calculated using two-tailed unpaired Student’s *t*-test (GraphPad Quick Calcs) by comparing treated and control groups with indicated number of biological replicates.

## Supporting information

S1 Fig Enhancement of YFV-induced IFN-β expression by BDAA isoforms with various antiviral potency. **(A)** Structure and antiviral activity of BDAA and its three isoforms. EC_50_ values were determined in experiments shown in panel B using GraphPad Prism 7. (**B**) HEK293 cells were infected with YFV at MOI of 0.01 for 1 h followed by treatment with indicated concentration of compounds. Total cellular RNA was extracted 48 hpi to detect YFV RNA and IFN-β mRNA by qRT-PCR. YFV RNA was expressed as percentage of untreated control. IFN-β mRNA was expressed as fold of induction relative to that in uninfected cells. Values represent average and standard deviation from 4 independent experiments. * indicates *P*<0.05, ** indicates *P*<0.01 (IFN-β enhancement relative to no treatment control).
(PDF)

S2 Fig Effect of YFV inhibitors on YFV RNA and IFN-β mRNA expression in HEK293/IFN-βLuc cells. 293/IFNβLuc cells were infected with YFV at MOI of 0.01 for 1 h followed by treatment with indicated concentration of BDAA (**A**), CGG-4088 (**B**), CGG-3394 (**C**), Sofosbuvir (**D**), or IHVR-19029 (**E**). Total cellular RNA was extracted at 48 hpi to detect YFV RNA and IFN-β mRNA by qRT-PCR. YFV RNA was expressed as percentage of YFV-infected and untreated control. IFN-β mRNA was expressed as fold of induction relative to that in uninfected cells. Values represent average and standard deviation from 3 independent experiments. * indicates *P*<0.05, ** indicates *P*<0.01 (IFN-β enhancement relative to no treatment control).
(PDF)

S3 Fig BDAA treatment enhances YFV-induced cytokine response in different cell types. HEK293 **(A)** HepG2 **(B)** and Huh7 **(C)** cells were infected with YFV at MOI of 10 for 1 h. At 24 hpi, cells were either mock treated with DMSO, or treated with 3μM of BDAA for 4 h. Total cellular RNA was extracted at 28 hpi. YFV RNA and indicated cytokine or chemokine mRNAs were detected by qRT-PCR and expressed as fold relative to that in uninfected cells. Values represent average and standard deviation from 3 independent experiments. * indicates *P*<0.05, ** indicates *P*<0.01, ***indicates *P*<0.001 compared to DMSO controls.
(PDF)

S4 Fig BDAA treatment induced exposure of dsRNA is sensitive to RNase III in HEK293 cells infected with YFV. HEK293 cells seeded on coverslips in 24-well plates were infected with YFV at MOI of 1 for 24 h followed by treatment with DMSO or 5μM BDAA for 6 h. **(A)** The cells were in situ permeabilized with Triton X-100, without or with RNase III treatment. **(B)** The cells were permeabilized by digitonin (Top row). Alternatively, after in situ permeabilization, cells were either additionally treated with RNase III (second row), RNase III treatment followed by permeabilization with Triton X-100 (third row), or RNase III treatment followed by permeabilization with Triton X-100 and another round of RNase III treatment (bottom row). Following the indicated treatment, the cells were fixed and incubated with YFV NS4B and dsRNA antibodies. YFV NS4B (red), dsRNA (green) and cell nuclei (blue) were visualized. Scale bar is 100μm.
(PDF)

S5 Fig YFV NS4B inhibitor CCG-4088 does not induce dsRNA exposure in digitonin permeabilized YFV-infected cells. Huh7 cells seeded on coverslips in 24-well plates were infected with YFV at MOI of 1. At 24 hpi, cells were treated with DMSO, 5 μM of CGG-4088 or 3 μM of BDAA for 6 h. The cells were then fixed and permeabilized with digitonin, followed by detection of YFV NS4B and dsRNA by immunofluorescence staining. NS4B (red), dsRNA (green) and cell nuclei (blue) were visualized. Scale bar is 100μm.
(PDF)

S6 Fig Characterization of YFV replication kinetics and interferon response in parental HEK293 and derived cell lines with RIG-I, MDA5 or MAVS knockout. The indicated cells seeded in 24-well plates were infected with YFV at MOI of 0.1. At the indicated time points, the cells were harvested, and total RNA was extracted. YFV RNA (**A**) and IFN-β mRNA (**B**) were measured by qRT-PCR and expressed as fold of induction relative to that in uninfected cells (n = 3).
(PDF)

S7 Fig Antiviral effect of BDAA in HEK293 and knockout cell lines infected with YFV. **(A)** Parental HEK293 and derived cell lines with the indicated gene KO cultured in 384-well plates were infected with YFV at MOI of 10 for 1 h, followed by treatment with the indicated concentrations of BDAA for 48 h. High-content imaging assay was performed to detect YFV NS4B protein (green). Cell nuclei were stained with DAPI (blue). Representative images are shown as a function of doses of BDAA treatment. **(B)** Percentage of cells with positive NS4B signal was expressed as average value based on multiple images taken from each well (n = 6).
(PDF)

S1 Table Summary of gene alterations in YFV-infected cells treated with BDAA. (DOCX)

S2 Table BDAA antiviral effect in HEK293 and knockout cell lines. (DOCX)

S3 Table qRT-PCR primers. (DOCX)
