# Supplementary figures and images for: A yellow fever virus NS4B inhibitor not only suppresses viral replication, but also enhances the virus activation of RIG-I-like receptor-mediated innate immune response
Source: PLoS Pathog. 2022 Jan 21;18(1):e1010271. doi: 10.1371/journal.ppat.1010271 (PMC8809586; doi:10.1371/journal.ppat.1010271)

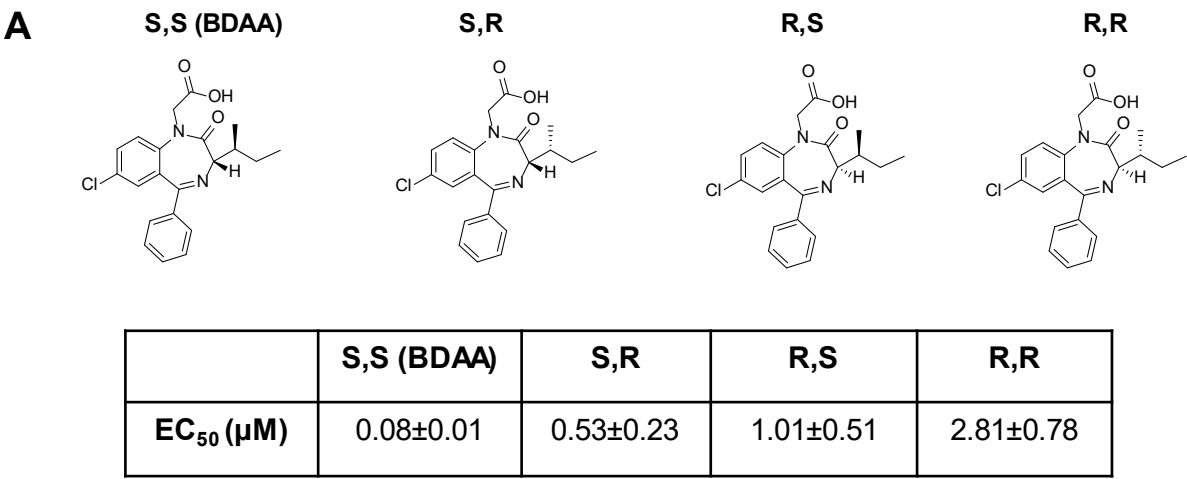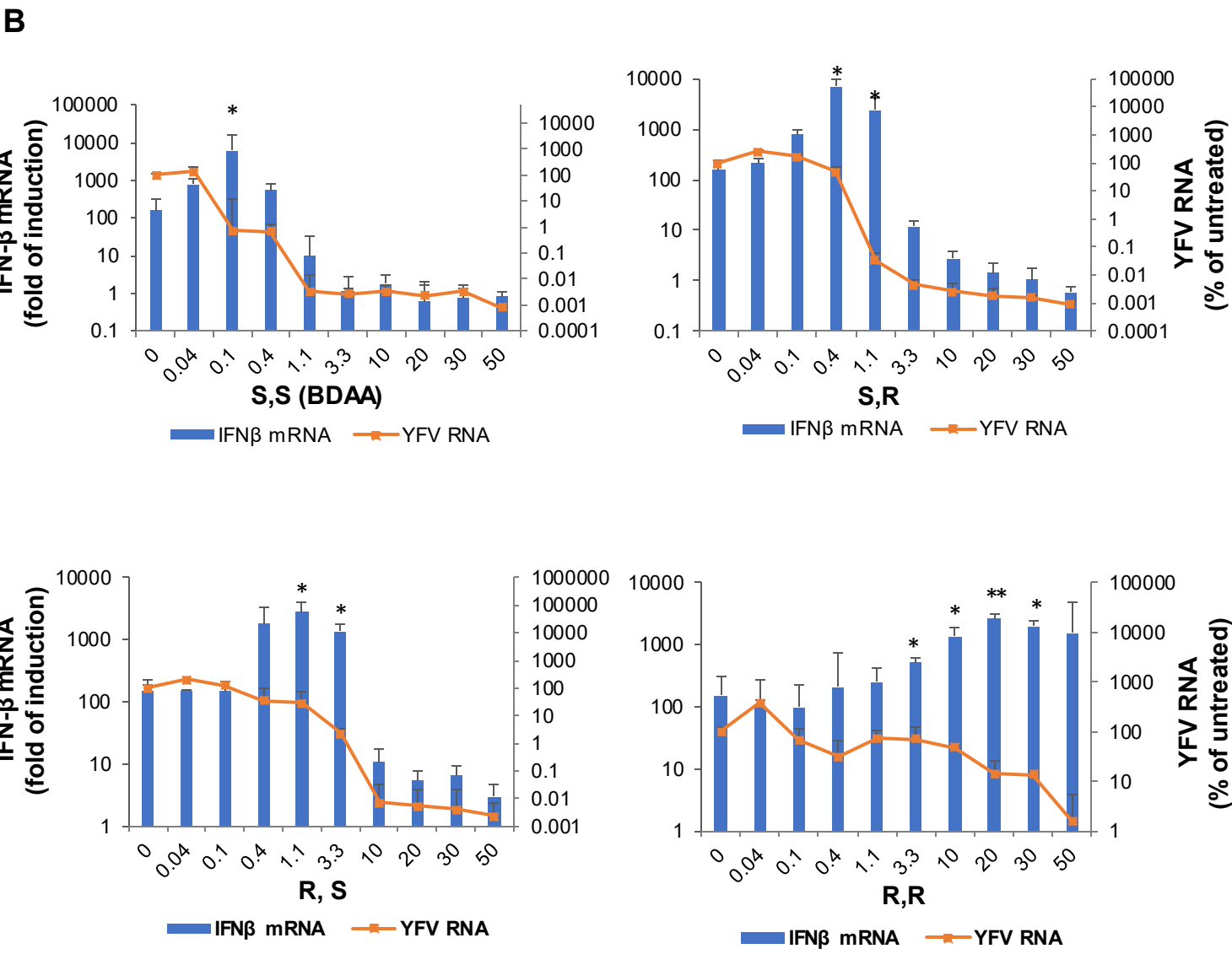

Supplemental Figure 1

Supplement: S1 Fig — (A) Structure and antiviral activity of BDAA and its three isoforms. EC50 values were determined in experiments shown in panel B using GraphPad Prism 7. (B) HEK293 cells were infected with YFV at MOI of 0.01 for 1 h followed by treatment with indicated concentration of compounds. Total cellular RNA was extracted 48 hpi to detect YFV RNA and IFN-β mRNA by qRT-PCR. YFV RNA was expressed as percentage of untreated control. IFN-β mRNA was expressed as fold of induction relative to that in uninfected cells. Values represent average and standard deviation from 4 independent experiments. * indicates P<0.05, ** indicates P<0.01 (IFN-β enhancement relative to no treatment control). (PDF) [file ppat.1010271.s001.pdf]

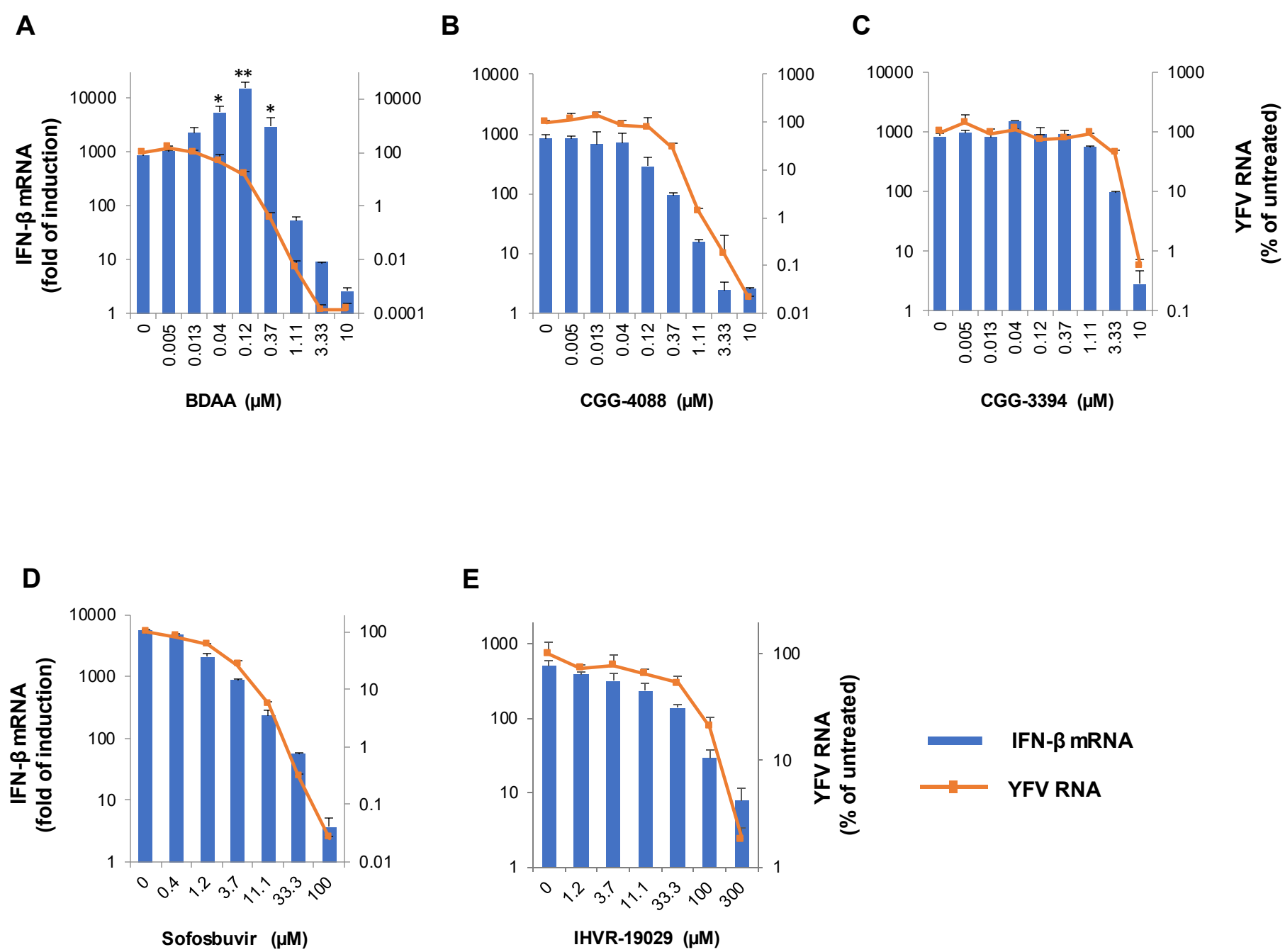

Supplemental Figure 2

Supplement: S2 Fig — 293/IFNβLuc cells were infected with YFV at MOI of 0.01 for 1 h followed by treatment with indicated concentration of BDAA (A), CGG-4088 (B), CGG-3394 (C), Sofosbuvir (D), or IHVR-19029 (E). Total cellular RNA was extracted at 48 hpi to detect YFV RNA and IFN-β mRNA by qRT-PCR. YFV RNA was expressed as percentage of YFV-infected and untreated control. IFN-β mRNA was expressed as fold of induction relative to that in uninfected cells. Values represent average and standard deviation from 3 independent experiments. * indicates P<0.05, ** indicates P<0.01 (IFN-β enhancement relative to no treatment control). (PDF) [file ppat.1010271.s002.pdf]

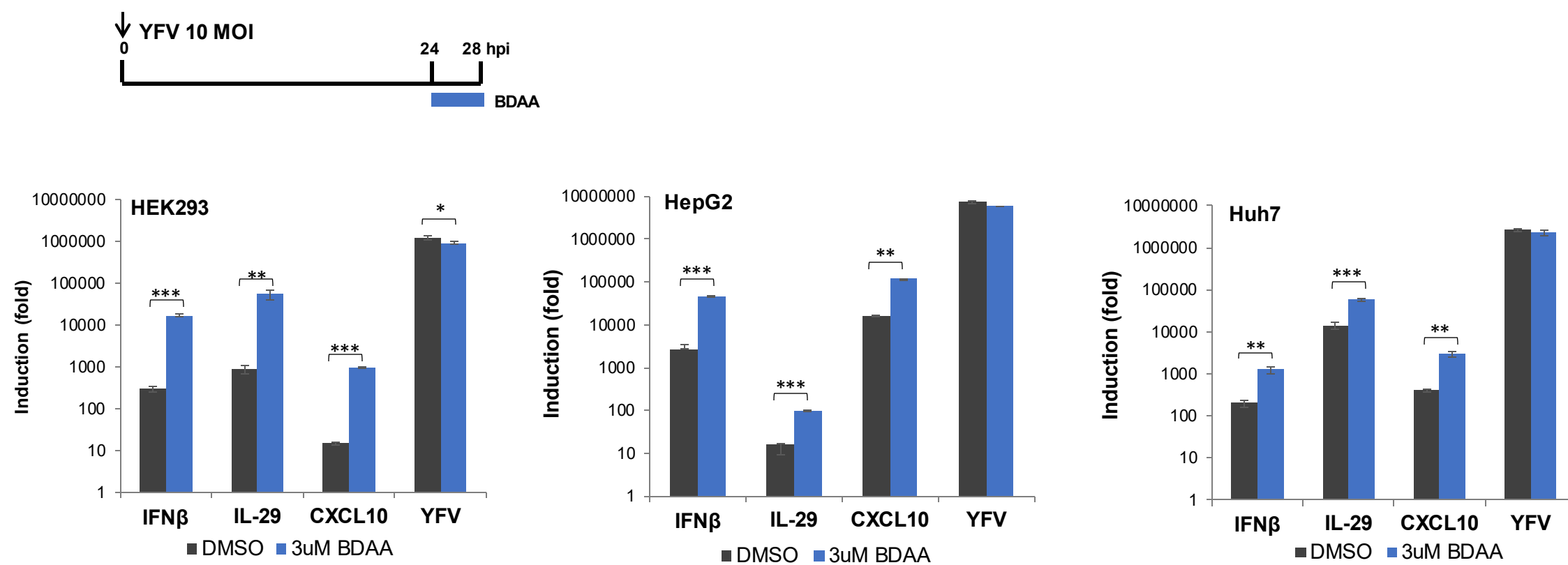

Supplemental Figure 3

Supplement: S3 Fig — HEK293 (A) HepG2 (B) and Huh7 (C) cells were infected with YFV at MOI of 10 for 1 h. At 24 hpi, cells were either mock treated with DMSO, or treated with 3μM of BDAA for 4 h. Total cellular RNA was extracted at 28 hpi. YFV RNA and indicated cytokine or chemokine mRNAs were detected by qRT-PCR and expressed as fold relative to that in uninfected cells. Values represent average and standard deviation from 3 independent experiments. * indicates P<0.05, ** indicates P<0.01, ***indicates P<0.001 compared to DMSO controls. (PDF) [file ppat.1010271.s003.pdf]

A

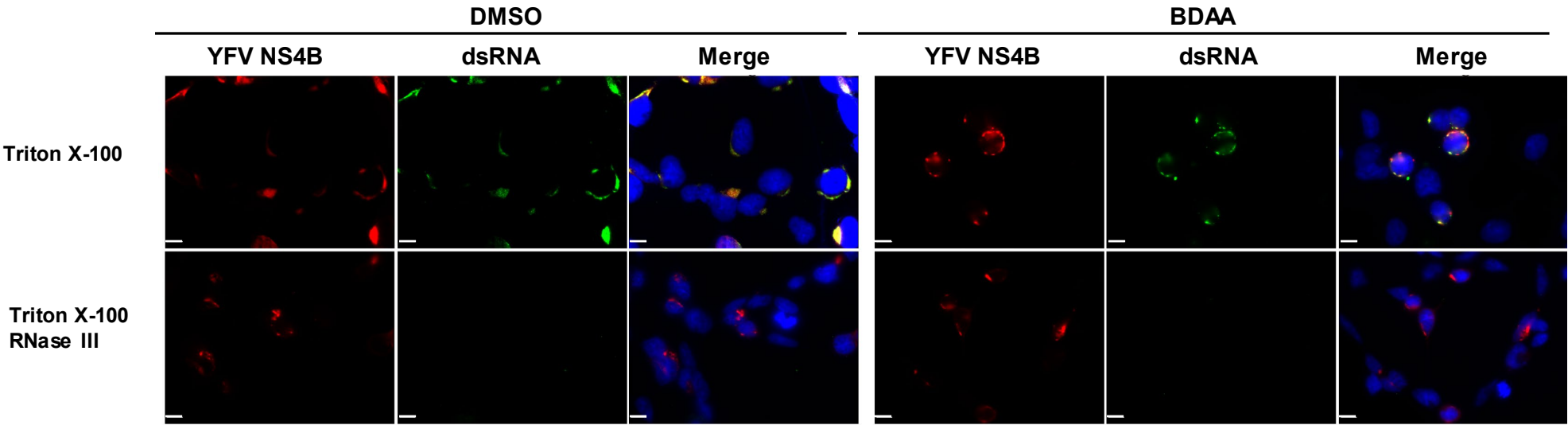

B

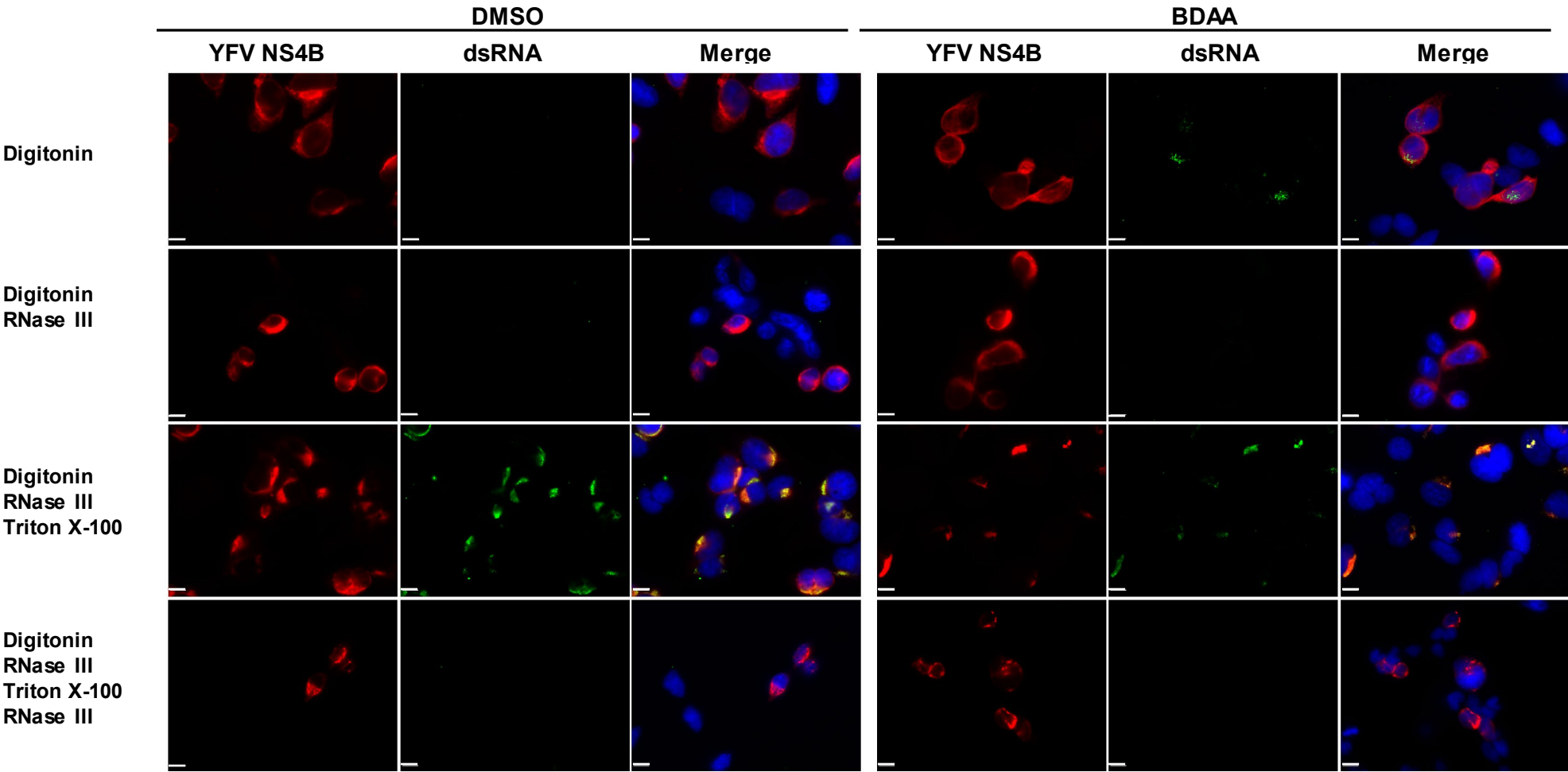

Supplemental Figure 4

Supplement: S4 Fig — HEK293 cells seeded on coverslips in 24-well plates were infected with YFV at MOI of 1 for 24 h followed by treatment with DMSO or 5μM BDAA for 6 h. (A) The cells were in situ permeabilized with Triton X-100, without or with RNase III treatment. (B) The cells were permeabilized by digitonin (Top row). Alternatively, after in situ permeabilization, cells were either additionally treated with RNase III (second row), RNase III treatment followed by permeabilization with Triton X-100 (third row), or RNase III treatment followed by permeabilization with Triton X-100 and another round of RNase III treatment (bottom row). Following the indicated treatment, the cells were fixed and incubated with YFV NS4B and dsRNA antibodies. YFV NS4B (red), dsRNA (green) and cell nuclei (blue) were visualized. Scale bar is 100μm. (PDF) [file ppat.1010271.s004.pdf]

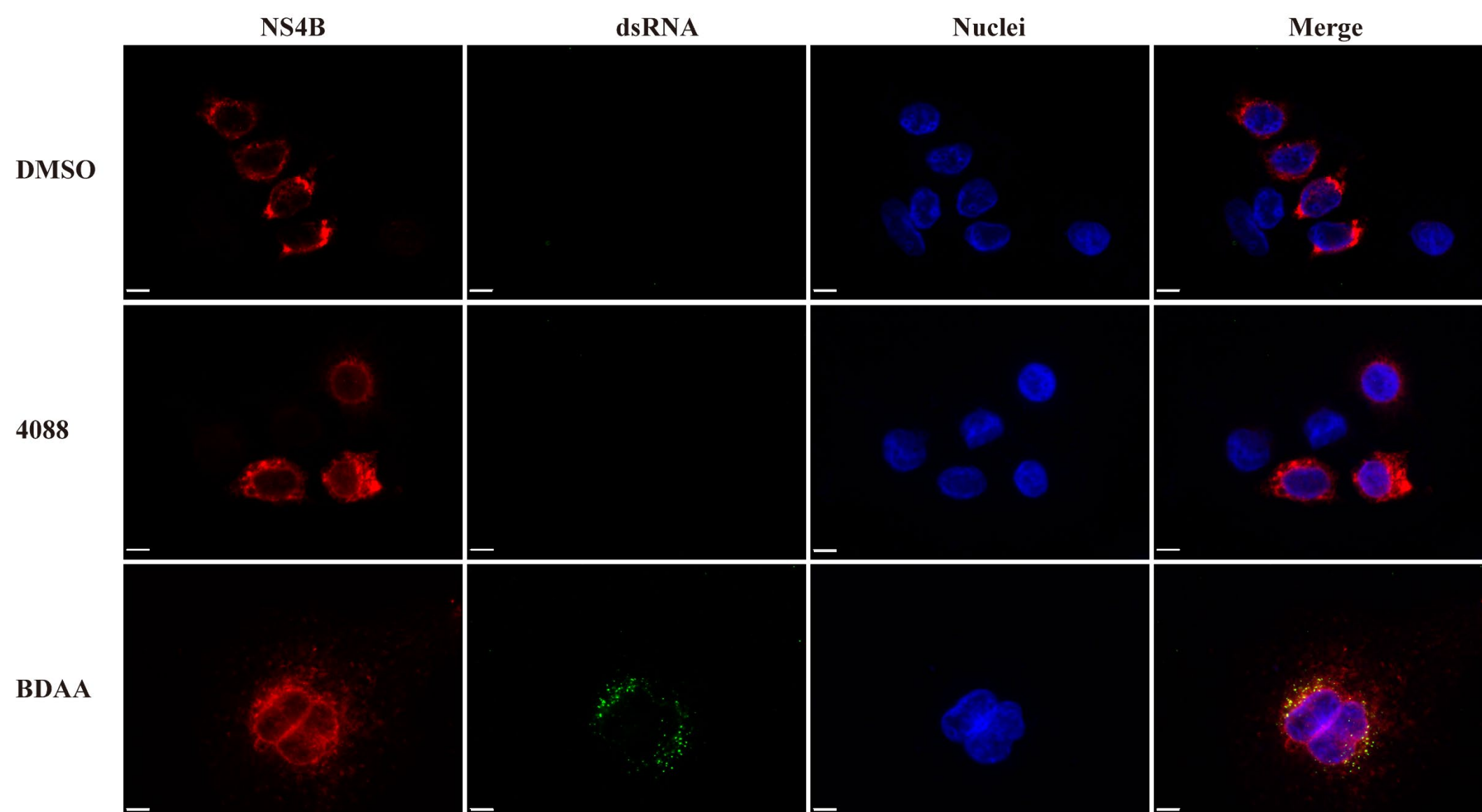

Supplemental Figure 5

Supplement: S5 Fig — Huh7 cells seeded on coverslips in 24-well plates were infected with YFV at MOI of 1. At 24 hpi, cells were treated with DMSO, 5 μM of CGG-4088 or 3 μM of BDAA for 6 h. The cells were then fixed and permeabilized with digitonin, followed by detection of YFV NS4B and dsRNA by immunofluorescence staining. NS4B (red), dsRNA (green) and cell nuclei (blue) were visualized. Scale bar is 100μm. (PDF) [file ppat.1010271.s005.pdf]

**A**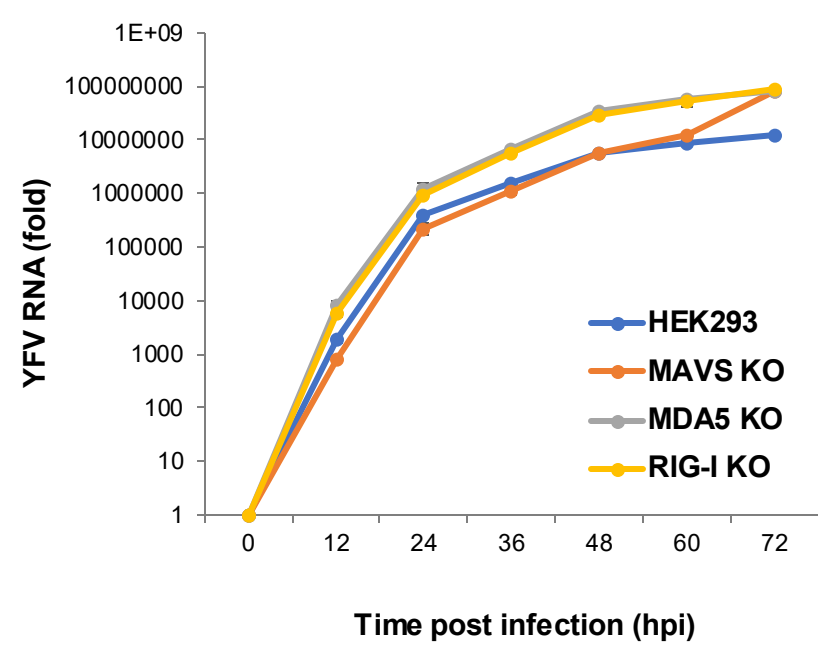**B**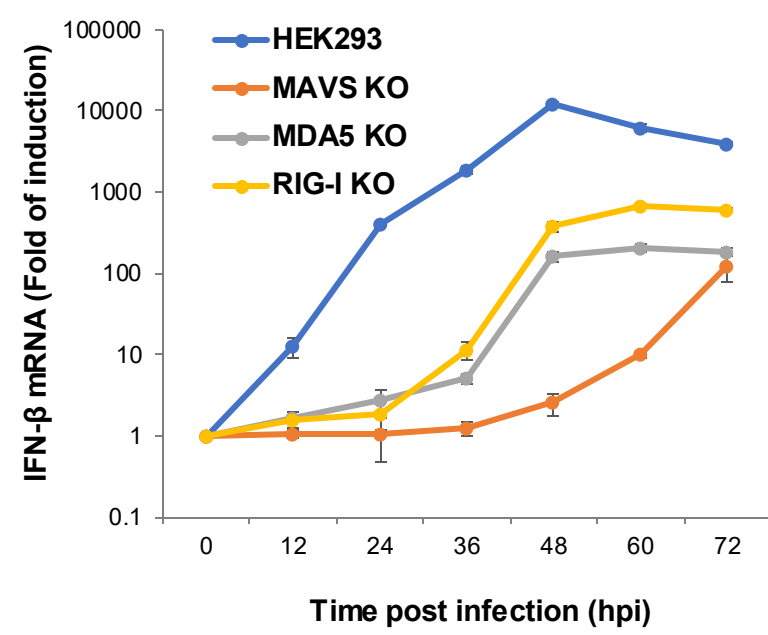

Supplemental Figure 6

Supplement: S6 Fig — The indicated cells seeded in 24-well plates were infected with YFV at MOI of 0.1. At the indicated time points, the cells were harvested, and total RNA was extracted. YFV RNA (A) and IFN-β mRNA (B) were measured by qRT-PCR and expressed as fold of induction relative to that in uninfected cells (n = 3). (PDF) [file ppat.1010271.s006.pdf]

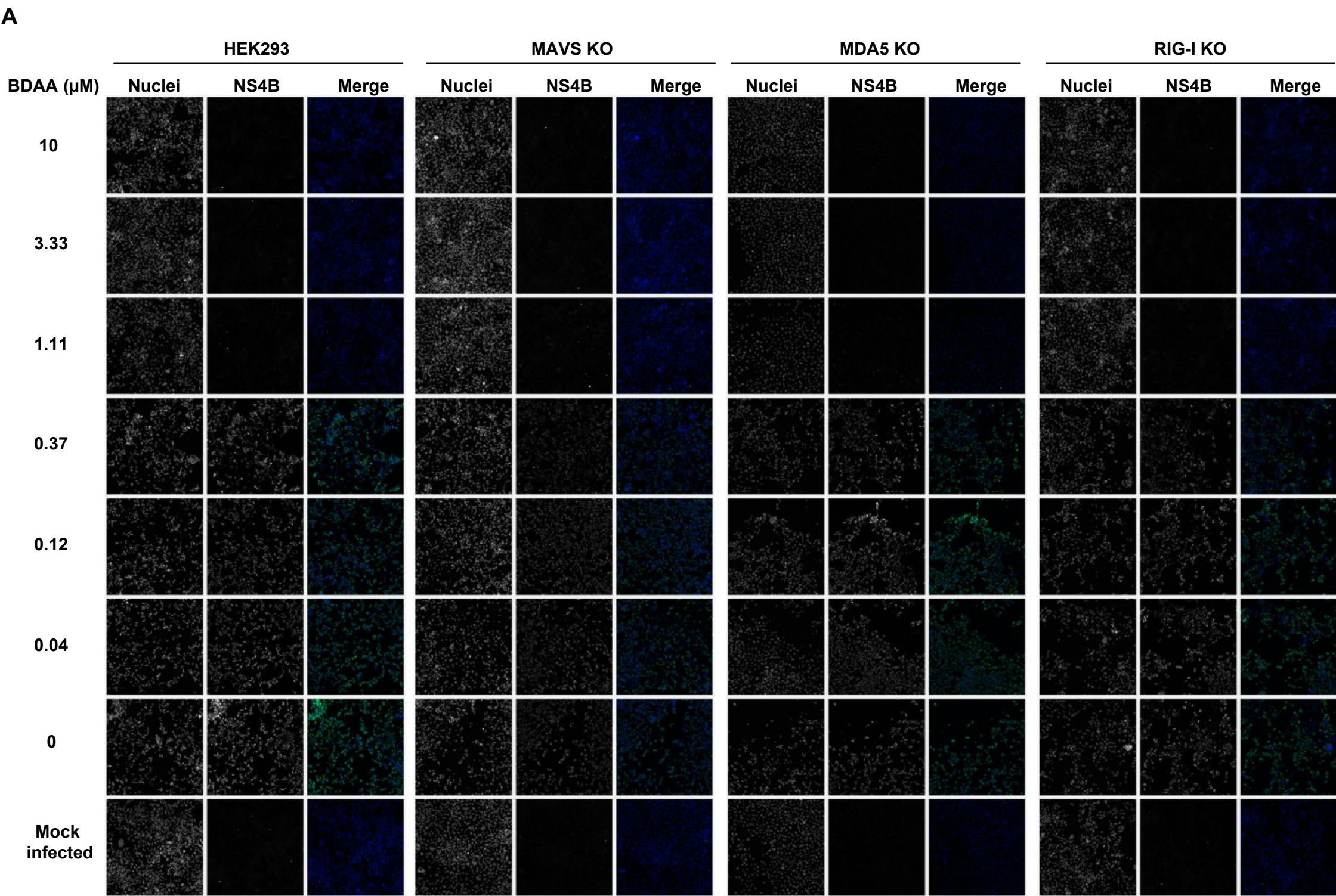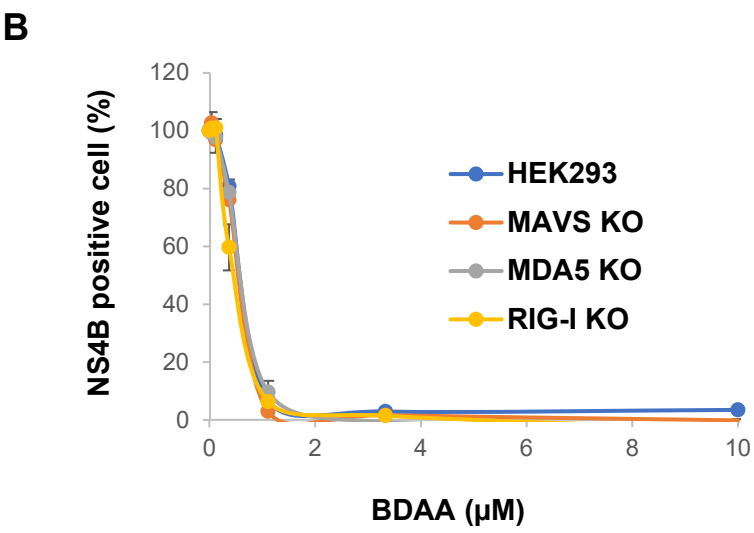

Supplemental Figure 7

Supplement: S7 Fig — (A) Parental HEK293 and derived cell lines with the indicated gene KO cultured in 384-well plates were infected with YFV at MOI of 10 for 1 h, followed by treatment with the indicated concentrations of BDAA for 48 h. High-content imaging assay was performed to detect YFV NS4B protein (green). Cell nuclei were stained with DAPI (blue). Representative images are shown as a function of doses of BDAA treatment. (B) Percentage of cells with positive NS4B signal was expressed as average value based on multiple images taken from each well (n = 6). (PDF) [file ppat.1010271.s007.pdf]
