# Supplementary material for: A yellow fever virus NS4B inhibitor not only suppresses viral replication, but also enhances the virus activation of RIG-I-like receptor-mediated innate immune response
Source: PLoS Pathog. 2022 Jan 21;18(1):e1010271. doi: 10.1371/journal.ppat.1010271 (PMC8809586; doi:10.1371/journal.ppat.1010271)
Supplement: S1 Table — (DOCX) [file ppat.1010271.s008.docx]

**S1 Table. Summary of gene alterations in YFV-infected cells treated with BDAA**

| **Category** | **Gene list** |
| --- | --- |
| Interferons  Other cytokines and chemokines  Interferon stimulated genes  Signal transduction and others | IFN-L1, IFN-L2, IFN-L3, IFN-A20P, IFN-B1, IFN-L4  CCL2, CCL5, CCL20, CXCL 1, CXCL2, CXCL3, CXCL8, CXCL10  ISG15, IFIT1, IFIT2, IFIT3, DDX58 (RIG-I), PMAIP1, GADD45B, TXNIP, PPP1R15A, CYR61, RND1, IER3, EGR1, ZFP36, OASL, CTGF, DUSP1, FOS, HES1  NFKBIA, NFKBIZ, TNF, TNFAIP3, MAP3K8, HSPA5 |

RNAseq parameters: FC>1.5, FDR<0.2. Underlined, down-regulated genes.
