# Supplementary material for: A yellow fever virus NS4B inhibitor not only suppresses viral replication, but also enhances the virus activation of RIG-I-like receptor-mediated innate immune response
Source: PLoS Pathog. 2022 Jan 21;18(1):e1010271. doi: 10.1371/journal.ppat.1010271 (PMC8809586; doi:10.1371/journal.ppat.1010271)
Supplement: S2 Table — (DOCX) [file ppat.1010271.s009.docx]

**S2 Table. BDAA antiviral effect in HEK293 and knockout cell lines**

| (µM) | **YFV infection (MOI=0.1)** | | | | **YFV infection (MOI=10)** | | | |
| --- | --- | --- | --- | --- | --- | --- | --- | --- |
|  | HEK293 | MAVS KO | MDA5 KO | RIG-I KO | HEK293 | MAVS KO | MDA5 KO | RIG-I KO |
| **EC_50_*** | 0.17±0.06 | 0.19±0.06 | 0.22±0.09 | 0.25±0.06 | 0.14±0.02 | 0.21±0.01 | 0.31±0.03 | 0.24±0.01 |
| **EC_90_*** | 0.40±0.16 | 0.52±0.15 | 0.32±0.09 | 0.42±0.04 | 0.35±0.03 | 0.61±0.01 | 0.74±0.01 | 0.67±0.09 |

*Determined by qRT-PCR assay and presented as average ± standard deviation (N=4).
