# Supplementary material for: A yellow fever virus NS4B inhibitor not only suppresses viral replication, but also enhances the virus activation of RIG-I-like receptor-mediated innate immune response
Source: PLoS Pathog. 2022 Jan 21;18(1):e1010271. doi: 10.1371/journal.ppat.1010271 (PMC8809586; doi:10.1371/journal.ppat.1010271)
Supplement: S3 Table — (DOCX) [file ppat.1010271.s010.docx]

**S3 Table. qRT-PCR primers**

|  | **Primers** |
| --- | --- |
| YFV RNA | forward: 5’-ATGCTGTCCCTTTTGGTTTG-3’  reserve: 5’-GCCACTGTGAGTTTCAGCAA-3’ |
| IFN-β | forward: 5’-ACTGCCTCAAGGACAGGATG-3’  reserve: 5’-AGCCAGGAGGTTCTCAACAA-3’ |
| IL28A | forward: 5’- TCGCTTCTGCTGAAGGACTGCA-3’  reserve: 5’- CCTCCAGAACCTTCAGCGTCAG-3’ |
| IL28B | forward: 5’- CTTTAAGAGGGCCAAAGATGC-3’  reserve: 5’- CCAGCTCAGCCTCCAAAG-3’ |
| IL29 | forward: 5’- TTCCAAGCCCACCACAAC-3’  reserve: 5’- TCCCTCACCTGGAGAAGC-3’ |
| TNFα | forward: 5’- CTCTTCTGCCTGCTGCACTTTG-3’  reserve: 5’- ATGGGCTACAGGCTTGTCACTC-3’ |
| CXCL10 | forward: 5’-GGTGAGAAGAGATGTCTGAATCC-3’  reserve: 5’-GTCCATCCTTGGAAGCACTGCA-3’ |
| IFIT1 | forward: 5’-ATCCAGGCGATAGGCAGAGATC-3’  reserve: 5’-GCCTTGCTGAAGTGTGGAGGAA-3’ |
| ISG15 | forward: 5’-CTCTGAGCATCCTGGTGAGGAA-3’  reserve: 5’-AAGGTCAGCCAGAACAGGTCGT-3’ |
| β-Actin | forward: 5’-CACCATTGGCAATGAGCGGTTC-3’  reserve: 5’-AGGTCTTTGCGGATGTCCACGT-3’ |
